# A Novel Clinical-Driven Design for Robotic Hand Rehabilitation: Combining Sensory Training, Effortless Setup, and Large Range of Motion in a Palmar Device

**DOI:** 10.3389/fnbot.2021.748196

**Published:** 2021-12-20

**Authors:** Raphael Rätz, François Conti, René M. Müri, Laura Marchal-Crespo

**Affiliations:** ^1^Motor Learning and Neurorehabilitation Laboratory, ARTORG Center for Biomedical Engineering Research, University of Bern, Bern, Switzerland; ^2^Force Dimension, Nyon, Switzerland; ^3^Department of Neurology, University Neurorehabilitation, University Hospital Bern (Inselspital), University of Bern, Bern, Switzerland; ^4^Department of Cognitive Robotics, Delft University of Technology, Delft, Netherlands

**Keywords:** robotic hand rehabilitation, clinical acceptability, neurorehabilitation, sensorimotor, haptics, clinical-driven, grasp, transparency

## Abstract

Neurorehabilitation research suggests that not only high training intensity, but also somatosensory information plays a fundamental role in the recovery of stroke patients. Yet, there is currently a lack of easy-to-use robotic solutions for sensorimotor hand rehabilitation. We addressed this shortcoming by developing a novel clinical-driven robotic hand rehabilitation device, which is capable of fine haptic rendering, and that supports physiological full flexion/extension of the fingers while offering an effortless setup. Our palmar design, based on a parallelogram coupled to a principal revolute joint, introduces the following novelties: (1) While allowing for an effortless installation of the user's hand, it offers large range of motion of the fingers (full extension to 180° flexion). (2) The kinematic design ensures that all fingers are supported through the full range of motion and that the little finger does not lose contact with the finger support in extension. (3) We took into consideration that a handle is usually comfortably grasped such that its longitudinal axis runs obliquely from the metacarpophalangeal joint of the index finger to the base of the hypothenar eminence. (4) The fingertip path was optimized to guarantee physiologically correct finger movements for a large variety of hand sizes. Moreover, the device possesses a high mechanical transparency, which was achieved using a backdrivable cable transmission. The transparency was further improved with the implementation of friction and gravity compensation. In a test with six healthy participants, the root mean square of the human-robot interaction force was found to remain as low as 1.37 N in a dynamic task. With its clinical-driven design and easy-to-use setup, our robotic device for hand sensorimotor rehabilitation has the potential for high clinical acceptance, applicability and effectiveness.

## 1. Introduction

With about 17 million people worldwide that experience a stroke each year, stroke remains a major cause of disability (Feigin et al., 2014). Up to 75% of stroke survivors suffer from long-term arm and hand impairments (Lai et al., 2002), which leads to a severe impact on patients' capability of performing activities of daily living and compromises their autonomy (Mercier et al., 2001; Hunter and Crome, 2002). To maximize recovery, clinical evidence suggests that patients should embark in active (Lotze, 2003), long (Kwakkel et al., 2004; Nielsen et al., 2015), high-intensity (Tollár et al., 2021), and repetitive functional task-specific practice (French et al., 2016). Sensory training is also highly recommended (Turville et al., 2019), as several studies have associated somatosensory impairment at baseline with poorer motor function and recovery after stroke (Meyer et al., 2014; Rowe et al., 2017). However, in practice, high-intensity therapy is labor-intensive, and training duration can be limited by the endurance and availability of the therapists, possibly reducing therapy outcomes. Furthermore, most of the current therapies target primarily improving motor functions, neglecting the sensory aspects of neurorehabilitation (Bolognini et al., 2016; Gassert and Dietz, 2018; Handelzalts et al., 2021).

The ideal neurorehabilitation training could be provided by robotic devices as robots can deliver high-intensity training in a motivating and engaging virtual environment (Brütsch et al., 2010; Lo et al., 2010; Gassert and Dietz, 2018; Bernardoni et al., 2019). However, despite the increasing number of robotic devices developed in the recent years for hand rehabilitation, the majority of these solutions has never been tested in clinical settings. One of the main obstacles listed for their poor clinical acceptance is high complexity—e.g., long setup times and overabundant functionalities (Balasubramanian et al., 2010). Furthermore, recent meta-analyses concluded that traditional robotic training yields similar or even inferior outcomes to conventional therapy, especially in activities of daily living (Bertani et al., 2017; Veerbeek et al., 2017). This is not surprising, since current rehabilitation robots only provide general assistance to perform rather artificial movements that are far from being functional. Current robot-aided interventions rely on abstract visual feedback while somatic (i.e., tactile and proprioceptive) feedback is underutilized. However, the perception of forces from the interaction with virtual environments conveys essential information for fine motor control and learning, e.g., during object grasping and manipulation (Huang et al., 2007; Danion et al., 2012; Özen et al., 2021). Thus, robots that enhance somatic information through haptic rendering—i.e., the provision of simulated interactive forces with virtual objects—might promote functional gains by leveraging practice in an enriched multisensory environment (Gassert and Dietz, 2018).

To evaluate the current state of the art on robotic hand rehabilitation, we performed an in-depth literature research and compared the found hand rehabilitation devices based on degrees of freedom (DoF), range of motion (RoM), available force, setup, and haptic rendering capabilities (see comparison table of hand rehabilitation devices in the Supplementary Material). Actuated hand rehabilitation devices can be distinguished in wearable exoskeletons, soft robotic gloves, grounded end-effectors, and grounded exoskeletons. The distinction between grounded exoskeletons and end-effector devices can be ambiguous in the case of hand rehabilitation devices, yet it is generally accepted that exoskeletons exert a high degree of control over individual joints and limb segments (Gassert and Dietz, 2018).

Wearable exoskeletons are usually mounted dorsally and often provide a large range of finger motion through sophisticated mechanisms that ensure coincident centres of rotation with the anatomical finger joints (Sarac et al., 2019). While some exoskeletons are principally designed to allow patients to perform rehabilitation exercises (e.g., Ho et al., 2011; Pu et al., 2020), others focus on assisting in activities of daily living (e.g., Hasegawa et al., 2008; Gasser et al., 2017; Hong et al., 2019). Because wearable exoskeletons tend to be cumbersome to install (Aggogeri et al., 2019), there has been an increasing effort to develop self-aligning (e.g., Zhang et al., 2014; Cempini et al., 2015; Leonardis et al., 2015; Sarac et al., 2016) as well as highly portable and mechanically simple exoskeletons [e.g., Tenoexo (Bützer et al., 2020), Mano (Randazzo et al., 2018)] to improve usability and ease of setup.

Similar to mechanically simple exoskeletons, soft-robotic gloves appear to be a promising alternative to complex exoskeletons for grasping assistance. They are often actuated by cables [e.g., CADEX (Kim and Park, 2018), Graspy Glove (Popov et al., 2017), CHAD (Huang et al., 2020), (Xu et al., 2020; Alnajjar et al., 2021)] or soft pneumatic actuators (e.g., Yap et al., 2016), which results in lightweight designs. Furthermore, they generally exhibit an excellent range of motion. The donning of soft robotic gloves has been facilitated by an open palm in the Glorea (Borboni et al., 2016) or a zipper on the palmar side of the glove in the BiomHED (Lee et al., 2014). Nevertheless, they require an advanced level of dexterity and finger mobility from the patients to be setup easily (Sarac et al., 2019).

While exoskeletons as well as soft robotic gloves create opportunities to integrate rehabilitation in activities of daily living, the vast majority of them is difficult to setup for patients suffering from compromised finger mobility due to spasticity or hypertonia (Tsai et al., 2019), which greatly limits the potential for interventions with these devices. Although a few wearable exoskeletons or soft robotic gloves are capable of haptic rendering [e.g., (Li et al., 2011; Sandoval-Gonzalez et al., 2016; Decker and Kim, 2017), CyberForce (CyberGlove Systems, USA), see Supplementary Material for further details], most of them do not yield this functionality. Due to their design, exoskeletons often allow to provide tactile sensory information by directly interacting with physical objects during exercises (e.g., Wang et al., 2018). Yet, the richness of sensory stimulation is limited to the properties of the physically available objects during therapy. Soft robotic gloves, on the other hand, generally have the disadvantage that the fingertips are covered, which might attenuate sensations from real-world object handling. Dedicated haptic devices, however, are able to provide rich sensory information with adjustable intensity, and importantly, can adapt continuously to the patients' specific needs and performance.

Several end-effector devices have been specifically developed to provide haptic feedback. The HIRO-III (Hioki et al., 2011) is a haptic interface that resembles a robotic hand with fingers that interact individually with the subject's fingertips. The underactuated orthosis of Sooraj et al., employs a three-bar linkage mechanism to individually actuate the fingertips and offers a large range of motion (Sooraj et al., 2013). Frisoli et al. proposed a high-fidelity haptic interface for thumb and index finger of the hand (Frisoli et al., 2007) with potential application in sensorimotor rehabilitation. The ReHapticKnob is a two DoF device (i.e., grasping and pronosupination) specifically designed to have excellent haptic rendering capabilities (Metzger et al., 2011). However, similar to the cable-driven HandCARE robot (Dovat et al., 2008) or the commercially available Amadeo^®^ (TyroMotion, Austria), the fingertips are attached to a linear axis and do not move along a physiological (i.e., spiral-shaped) finger path. The reachMAN2 is a haptic device for reach and grasp training with a palmar handle—i.e., the handle is largely in contact with the palmar side of the hand. The Alpha-Prototype II (Masia et al., 2007) is a palmar robotic handle with an axially symmetrical design capable of high-quality haptic rendering. It is—similar to other palmar end-effector devices [e.g., (Just et al., 2019), InMotion^®^ Arm/Hand (Bionik Labs, Canada)]—relatively simple to setup. However, current palmar devices generally suffer from a limited range of motion.

When it comes to grounded hand exoskeletons, Ueki et al. developed a device for hand and wrist rehabilitation that controls 18 DoF (Ueki et al., 2012) employing dedicated linkage mechanisms for each finger. The HEXORR, developed by Schabowsky et al. (2010), is a grounded robotic exoskeleton which implements simultaneous movements of index to little finger with a large range of motion. The FINGER exoskeleton (Taheri et al., 2014) is highly backdrivable and can be used for proprioceptive training of two fingers. The hand module Manovo® Power (Hocoma, Switzerland) offers one DoF (i.e., coupled finger and thumb motion), uses straps for an easy setup, but provides only limited finger flexion. Finally, The Gentle/G hand module (Loureiro and Harwin, 2007) provides basic haptic rendering and allows to interact with virtual environments. It is equipped with a hinge mechanism that allows to open the hand fixations for a quick setup.

Based on the reviewed studies, there is a clear need for a new actuated hand rehabilitation device that is easy to setup while allowing for a large range of finger motion, and that provides physical assistance as well as somatic sensations to practice meaningful functional tasks in an engaging virtual environment. To address the unsatisfied needs in robotic sensorimotor rehabilitation, we aimed at developing a novel clinical-driven robotic hand rehabilitation device that is capable of high quality haptic rendering and that supports physiological full flexion/extension of the fingers while offering an effortless setup.

**Figure 1:**
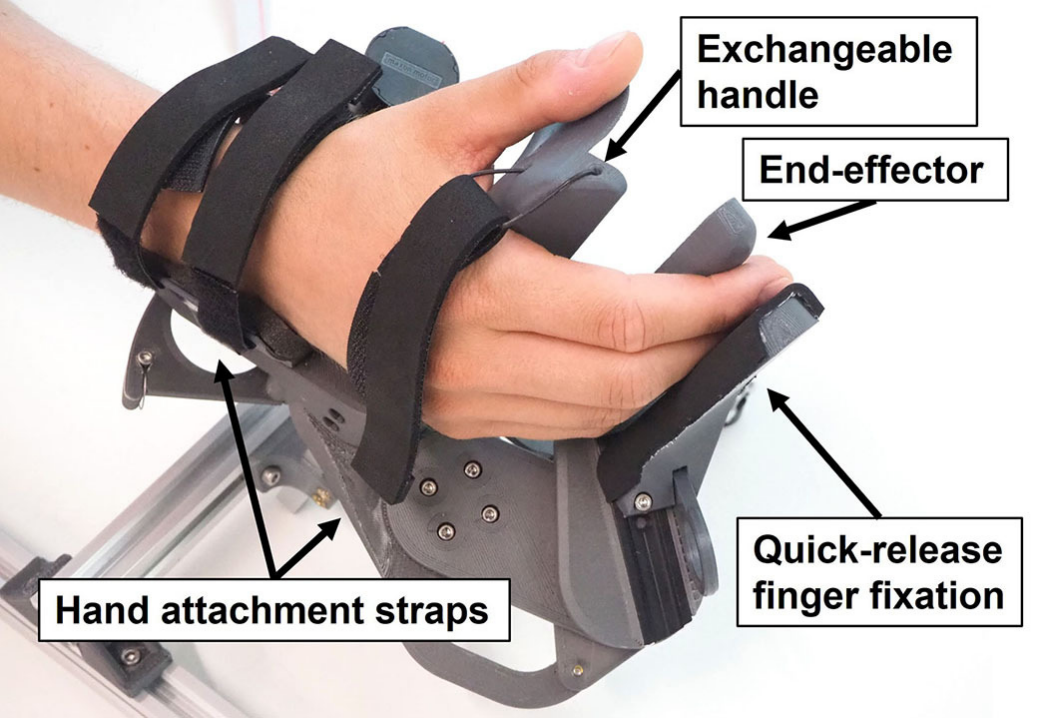
Prototype of the clinical-driven Palmar RehabilitatIon DEvice (PRIDE).

To maximize acceptance and usability of our novel device, we conducted a survey with 33 participants (therapists, nurses, and physicians working in neurorehabilitation) to gather clinical requirements (Rätz et al., 2021). The results from this survey confirmed that a simple and short setup is essential for the clinical acceptability and applicability of robotic devices in rehabilitation. Furthermore, finger extensions were reported as crucial movements to be trained. To fulfill these clinical requirements, we combined novel optimization methods that incorporate not only mechanical considerations (i.e., simple setup, fine haptic capabilities, accommodating diverse hand sizes), but importantly, also anatomical considerations (i.e., large physiological range of motion, different lengths of individual fingers, ergonomic grasp). Here, we present the resulting optimal design, the Palmar RehabilitatIon DEvice (PRIDE) (Figure 1), which introduces the following novelties:

1. A large range of motion (from 180° flexion to full extension) of the fingers, while allowing for an effortless installation of the patient's hand. This is achieved by designing the handle to have a compact cylindrical shape during the setup phase.
2. Our kinematic design ensures that all fingers are supported through the full range of motion and that the little finger does not lose contact with the handle during extension.
3. In our design, we took into consideration that the human hand usually grasps a cylindrical object in a way that it is not orthogonal to the longitudinal axis of the hand. Instead, it runs obliquely from the metacarpophalangeal joint of the index finger to the base of the hypothenar eminence.
4. The end-effector path was optimized to guarantee physiologically correct finger movements for a large variety of hand sizes.

In the upcoming sections, we present the requirements as well as the mechanical design and control of our prototype. First, the requirements are established based on clinical needs, anatomical constraints, and mechanical considerations. A kinematic design that satisfies all the requirements is then proposed and optimized based on anthropometric data. The mechanical realization and the control thereof are then described, including friction and gravity compensation to enhance transparency. Finally, we present results from a preliminary test with healthy participants to characterize the device's haptic capabilities.

## 2. Methods

### 2.1. Requirements

#### 2.1.1. Clinical Requirements

**Figure 2:**
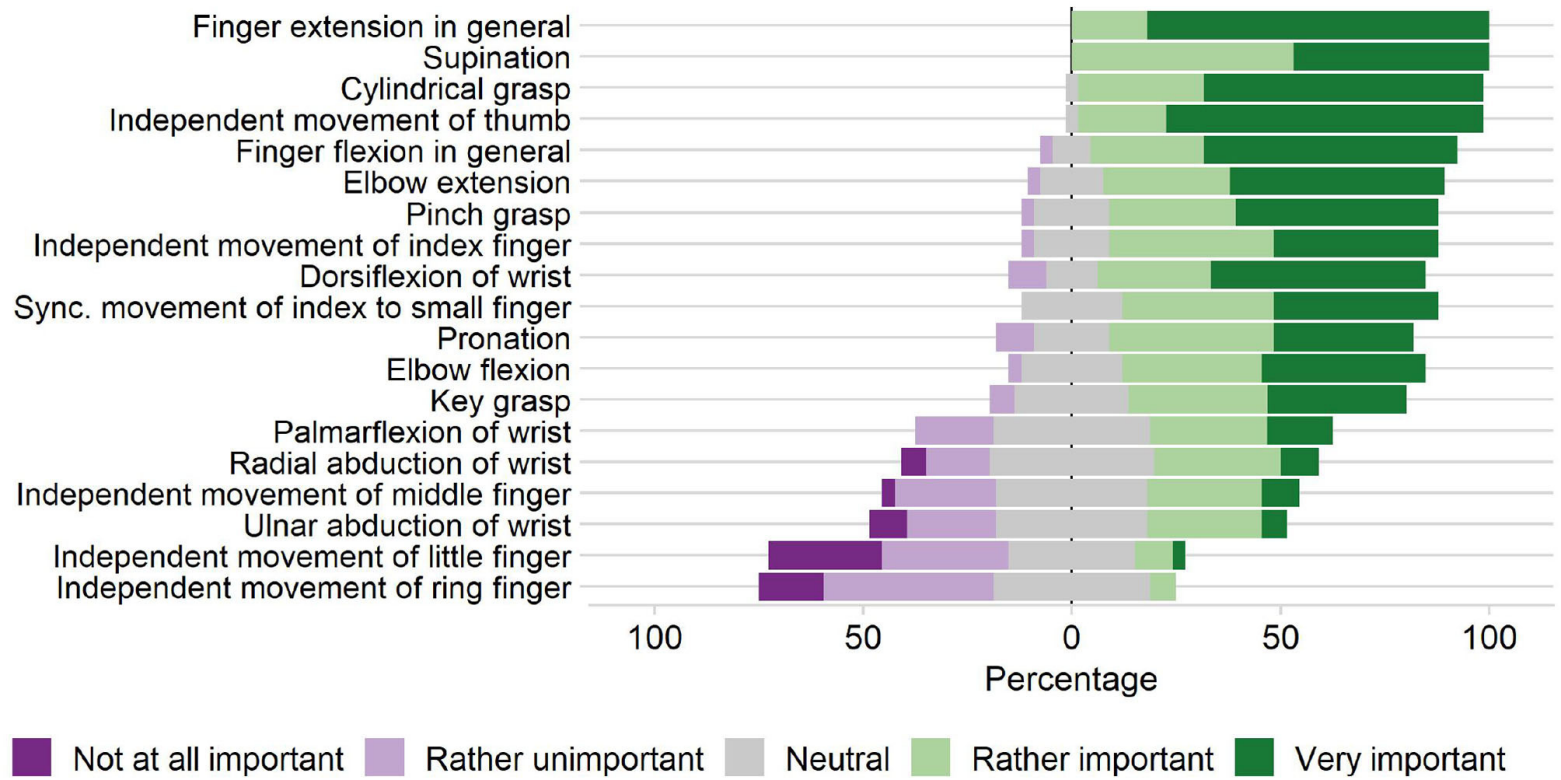
Clinical requirements. Importance of practicing various upper-limb movements in stroke rehabilitation according to our survey with 33 participants (therapists, nurses, and physicians working in neurorehabilitation) from the University Hospital Bern, Switzerland and Reha Rheinfelden, Switzerland.

Prior to the novel device development, we conducted a survey with 33 clinical professionals (therapists, nurses, and physicians working in neurorehabilitation) from the University Hospital Bern, Switzerland and Reha Rheinfelden, Switzerland, to gather the clinical requirements for a robotic device targeting sensory-motor rehabilitation of the upper-limbs (Rätz et al., 2021). We found that grasping, eating, and personal hygiene are amongst the most important activities of daily living to be exercised. Finger and wrist extensions were reported as relevant movements to be trained. In subsequent on-site discussions with therapists during the device development, we further particularized full finger extensions as a crucial clinical requirement. Moreover, the results of our survey indicated a higher relevance of training the index finger compared to middle, ring and small finger (Figure 2). Importantly, the majority of the clinicians would like to spend less than 10 min (median of 5 min) to set up the robotic device. A complete list of the survey results can be found in Rätz et al. (2021).

#### 2.1.2. Anatomical Requirements

##### Finger Model and Interjoint Couplings

**Figure 3:**
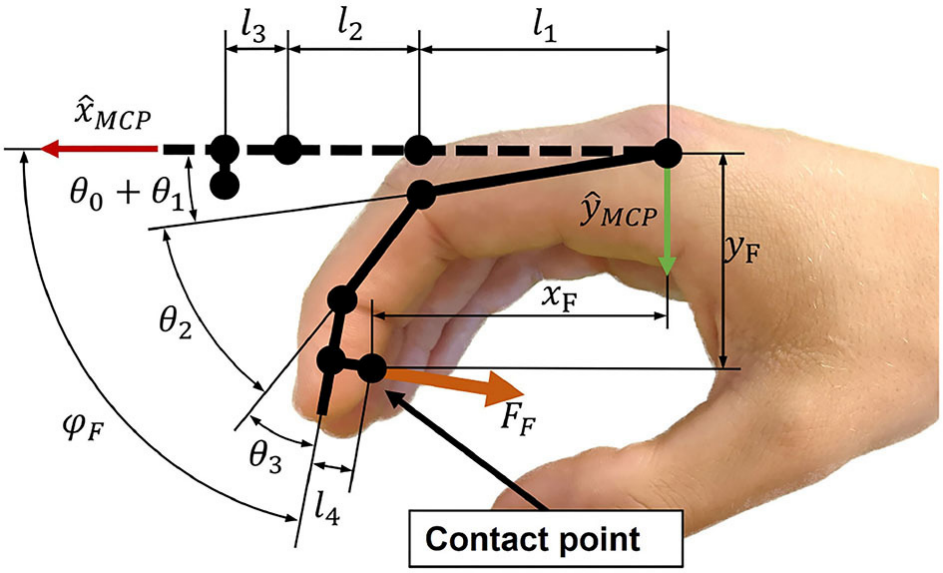
Schematic representation of the index finger kinematics.

To support physiological finger movements, we first need to understand the path that is described by a finger in a grasping motion. To this end, a kinematic model of the fingers is required. We utilized the three DoF kinematic model depicted in Figure 3. The metacarpophalangeal (MCP; θ_1_), proximal interphalangeal (PIP; θ_2_) and distal interphalangeal (DIP; θ_3_) joints were considered as one DoF hinge joints. The angle θ_0_ denotes the initial angle of the MCP joint. The abduction and adduction of the fingers were assumed to be zero. Further, all joints axes were assumed to be parallel to ẑ_*MCP*_, which constrains the finger movements to be within the *xy*-plane (Figure 3). The finger tip angle w.r.t. to the metacarpal bone is denoted as φ_*F*_. The fingertip position coordinates *x*_*F*_ and *y*_*F*_ as well as φ_*F*_ are computed by Equation (1), whereby *c*_01_ is the short form of cos(θ_0_ + θ_1_), etc.


(1) $$\begin{array}{l} x_{F}=l_{1}c_{01}+l_{2}c_{012}+l_{3}c_{0123}-l_{4}s_{0123}\\ y_{F}=l_{1}s_{01}+l_{2}s_{012}+l_{3}s_{0123}+l_{4}c_{0123}\\ \varphi_{F}=\overset{3}{\underset{i=0}{\sum }}\theta_{i} \end{array}$$

We assumed that consecutive finger joint positions can be described as a function of the preceding finger joints. These interjoint couplings *n*_12_ and *n*_23_ were defined as follows:


(2) $$\begin{array}{l} \theta_{2}=n_{12}\theta_{1}\\ \theta_{3}=n_{23}\theta_{2}=n_{12}n_{23}\theta_{1} \end{array}$$

**Figure 4:**
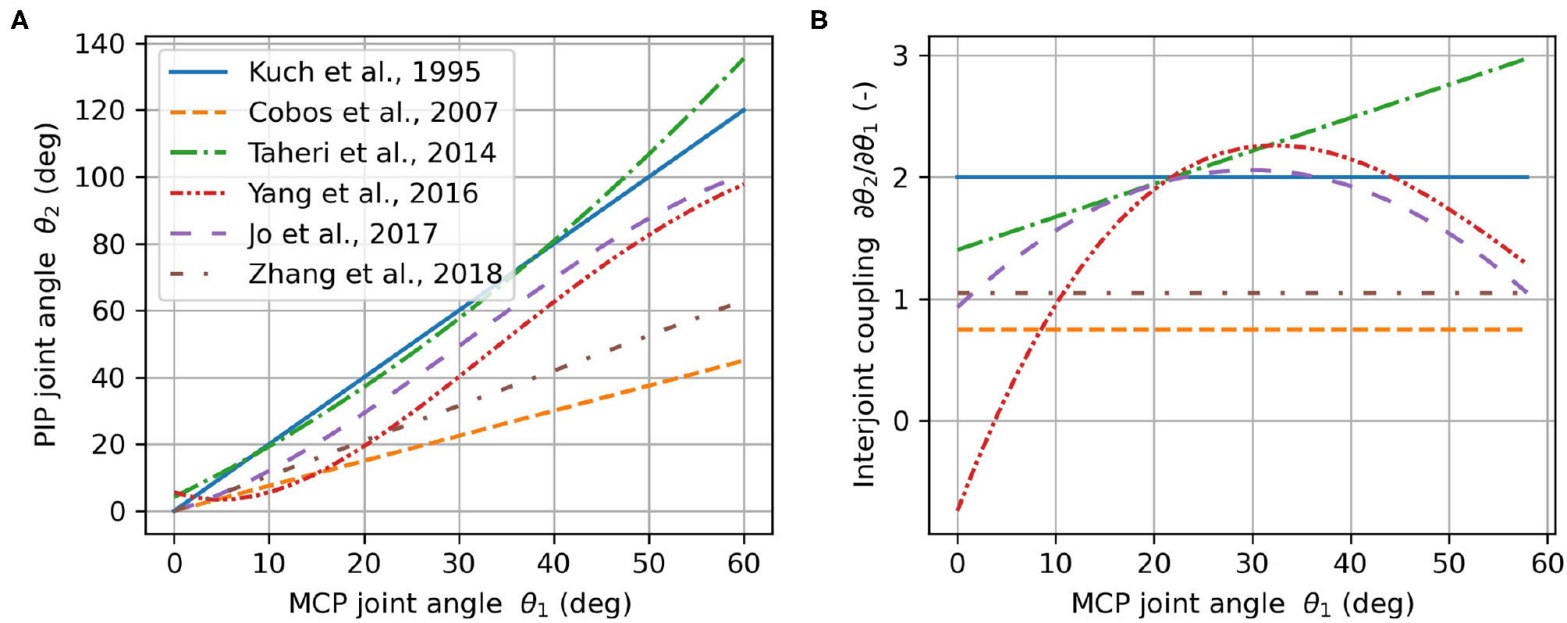
Angular relationship between PIP and MCP joints **(A)** and the corresponding interjoint coupling (rate of change) **(B)** according to literature.

It is generally accepted that there is an approximately constant anatomical coupling between the DIP and PIP joints that lies in the range of *n*_23_ ∈ [0.65, 0.75] for the index finger (Hahn et al., 1995; Cobos et al., 2007; Mentzel et al., 2011) while the movements of the MCP and PIP joints are independent to each other. It is, for example, possible to fully flex the PIP joint while extending the MCP joint and vice-versa. The typical relation of the MCP and PIP joint angles *n*_12_ during grasping for healthy individuals has been subject to investigation and has been described as linear (Kuch and Huang, 1995; Cobos et al., 2007; Zhang et al., 2018), quadratic (Taheri et al., 2014), cubic (Jo et al., 2017), or even quartic (Yang et al., 2016) (Figures 4A,B). Based on this large and inconsistent variety of identified MCP-PIP interjoint couplings, we argue that a constant coupling between MCP and PIP joint angles can be assumed, which results in a physiologically correct and comfortable grasping motion. For the development of our prototype, the value of this constant coupling was chosen to be in the range of *n*_12_ ∈ [1.25, 1.75]. This allows to reduce the three DoF finger model in Equation (1) to a one DoF model.

##### Cylindrical Grasping

**Figure 5:**
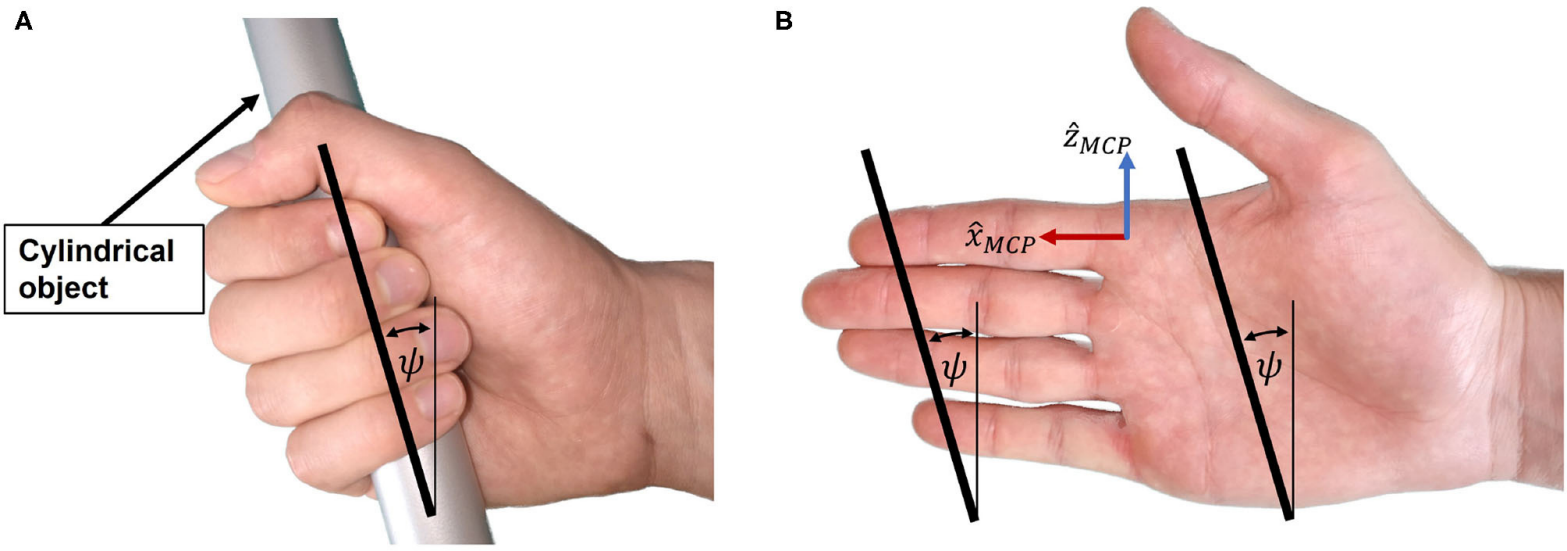
Cylinder angle. **(A)** The hand encloses the cylindrical object with the cylinder angle. **(B)** Illustration of the cylinder angle in the open hand as well as the line connecting the fingertips from index to little finger.

When holding a cylindrical object using full palmar prehension, the longitudinal axis of the cylindrical object usually runs obliquely from the MCP joint of the index finger to the base of the hypothenar eminence (Napier, 1956). We refer to the angle of this longitudinal axis relative to the transverse axis of the hand in Figure 5A as *cylinder angle*, as introduced by Buchholz (Buchholz, 1992). A wide range of cylinder angles, from 10° to 30°, has been reported in literature (Kapandji, 1982; Buchholz, 1992).

If a straight line is traced in the coronal plane through the center of the distal segment of the index finger and the center of the distal segment of the little finger (Figure 5B), the angle between this line and the transverse axis of the hand happens to be similar to the cylinder angle ψ. Importantly, this is also the case if the same line is traced when the hand is closed (Figure 5A). The straight line connecting the centers of the distal segments of index and little finger appears to be parallel to the longitudinal axis of a cylindrical object in a cylindrical grasp with flexed (Figure 5A) or extended (prior to grasping, Figure 5B) fingers. This is due to a combination of several factors: First, the length of each finger is different, with the little finger being the shortest. Second, the MCP joint of each finger has a different proximodistal position with the MCP joint of the little finger being the most proximal (Vergara et al., 2018). Third, as described by Kapandji (Kapandji, 1982), the last three fingers—i.e., middle finger, ring finger and little finger—not only move in the sagittal plane when flexed, but in an oblique plane latero-medially, with the small finger moving in the most oblique plane. Last, the increased functional range of motion of the MCP joints of ring and little finger (Hayashi and Shimizu, 2013) bring their fingertips in a more proximal position in a cylindrical grasp.

Because of the apparent resemblance of the cylinder angle and the angle deduced from the line through the fingertips of index and little finger, we assume these angles to be equivalent and denote them both by ψ as indicated in Figure 5B. To enable a natural cylindrical grasp, we require our kinematic design to respect this angle, which we assumed to be ψ = 25° based on estimations of the fingertip positions of index and little finger using (Garrett, 1970a,b; Vergara et al., 2018).

#### 2.1.3. Design Requirements

##### Easy Setup

After stroke, patients often suffer from spasticity (Urban et al., 2010), leading to involuntary chronic joint flexion. This presents an insuperable barrier for the usage of devices that require finger extension during setup. Thus, to facilitate the admission of patients with spasticity into robotic rehabilitation, it would be advantageous to perform the patients' setup with their hands being closed. We, therefore, aimed at designing a device that possesses a compact, cylindrical-shaped geometry during setup, which allows to slide the patient's closed fist on the device.

The simultaneous clinical requirement of being able to practice full finger extension movements and the design requirement of a compact handle to facilitate the setup, impose the need of a large range of motion of the fingers. We determined a fingertip full flexion angle φ_*F*_ of 180° as a reasonable value for a closed hand and φ_*F*_ of 0° at fingers full extension. This choice represents a compromise between compactness of the handle during setup, full finger extension, and mechanical feasibility.

##### Fingertip Forces During Grasping

Including all digits, the human hand is capable of approximately 500 N grasping force (100 N for the thumb during a key grasp) (Hasser, 1995; Rickert, 2010). Wiker et al. report that a value of 15% of the maximum voluntary finger contraction, corresponding to 75 N in the fingertips, is an upper bound for the comfortable long-term use of a haptic interface (Wiker et al., 1989). A lower bound can be deducted from the peak force that is required to extend moderately spastic/hypertonic fingers. In literature, values ranging from approximately 15 N (Kamper et al., 2006) to 25 N, (15 N for the thumb) (Nycz et al., 2018) are reported. Thus, a minimum continuous fingertip force of *F*_*F,min*_ = 30 N was considered as an adequate value for the development of our haptic device. The force during practice was decided to be applied on the last segment of the fingers, which corresponds to natural grasping. This would further promote sensory stimulation at the fingertips where the highest density of cutaneous mechanoreceptors is located (Vallbo and Johansson, 1984).

##### Accounting for Different Hand Sizes

In order to increase the clinical practicability of our solution, we aimed to design a device that could be used by patients with a variety of hand proportions, i.e., from the 5th percentile of women's hand size to the 95th percentile of men's hand size. Exhaustive measurements of hand proportions are reported in the NASA Man-System Integration Standards (NASA, 1995), in the studies of Garrett (1970a,b) and by Vergara et al. (2018). Buchholz et al. represent the length of each finger segment as a percentage of the total hand length (Buchholz et al., 1992). Their estimations agree with the findings of Van Der Hulst et al. (2012). For our development, we utilized the values reported by Garrett and Buchholz et al., and we assumed that all the proportions of finger segments scale linearly with the hand size.

##### Enhanced Transparency

Haptic interactions can be finely rendered without the need of adding expensive/bulky force sensors by employing open-loop impedance control (Hatzfeld and Kern, 2014). Yet, this requires that our mechanical design is inherently transparent, i.e., possesses low static friction, low backlash, and high backdrivability. A highly backdrivable design also has the advantage of being inherently safe in case of a power cut-off. Cable-driven transmissions could be good candidates to achieve high transparency and are already successfully employed in many haptic devices (e.g., Mali and Munih, 2006; Pezent et al., 2017; Buongiorno et al., 2018).

### 2.2. Mechanical Design

In our solution, we employ a palmar design—i.e., the main area of contact between the device and the hand is between the handle and the palm (Figure 1). While the metacarpal bones of the hand are fixed on the handle using straps, the fingertips are attached to an end-effector, which moves the fingertips along a specific path. In this paper, we refer to the *end-effector* as the part of our device which interacts with the user's fingertips. While a large part of the distal finger segments might touch the end-effector, the contact point between a fingertip and the end-effector is defined to be in the center of the palmar side of the distal finger segment (Figure 3). After finding an adequate kinematic architecture for our device based on clinical, anatomical and design requirements, the synthesis of the mechanical design parameters was performed utilizing an optimization approach (see section *Optimization of Design Parameters*). The key idea of this optimization was to find the position of hands of different sizes on the device such that their respective fingertip paths overlap when performing a cylindrical grasp.

**Figure 6:**
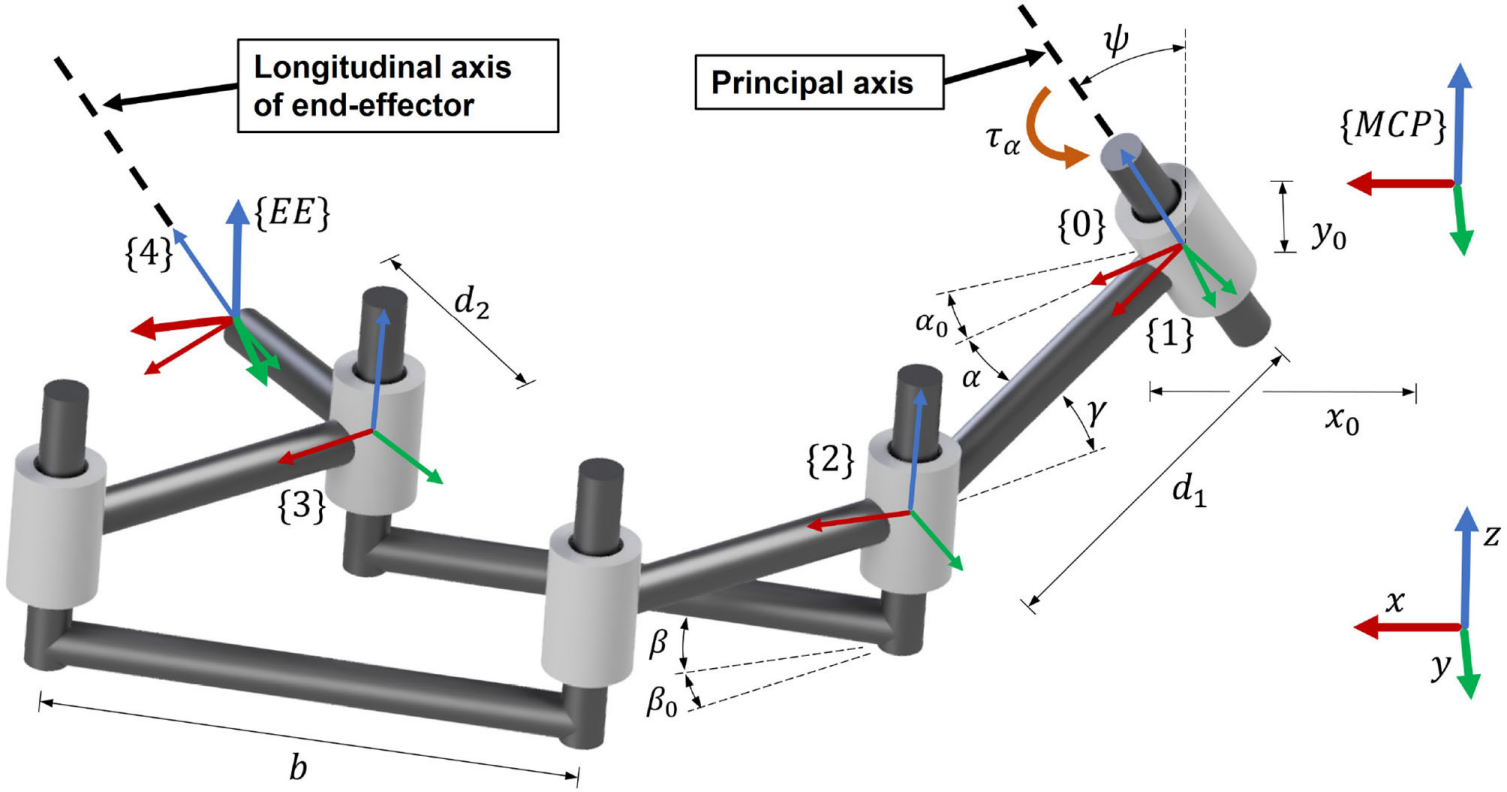
Schematic representation of the kinematics of the device.

Motivated by the results of our survey, which highlights the relevance of training natural index finger movements (Rätz et al., 2021), the kinematic design was realized focusing on the index fingertip path. When performing a cylindrical grasp, the fingertips approximately describe a spiral around the MCP joints, which consists of a rotary movement around a principal revolute axis with a successively decreasing momentary radius. To accomplish that the robot end-effector follows this spiral-like movement, a parallelogram coupled to a principal revolute joint was chosen (Figure 6). Within this design, the parallelogram moves as a function of the rotation α of the principal revolute joint, and hence, requires only one actuator. Furthermore, this solution uses solely revolute bearings, which typically possess lower friction values and require less maintenance than linear bearings because their races are less exposed and can be more easily protected from dust than the rails of linear bearings.

To achieve a natural cylindrical grasp, the cylinder angle ψ (Figure 5) was introduced into the system design. We found that a remarkably natural grasping motion can be obtained when the orientation of the handle's longitudinal axis (corresponding to the orientation of a straight line connecting the centers of the fingertips of index and little finger as shown in Figure 5) is invariant to the world frame during the entire range of motion. In particular, this allows the small finger to stay in contact with the end-effector and avoids that it slides off the end-effector during the entire range of motion. Thus, we tilted the entire mechanism forwards, including the principal (actuated) revolute joint, with an angle ψ (Figure 6) w.r.t. the transverse axis of the hand. The fingertip support (end-effector) was then designed to be parallel to this tilted principal axis of rotation in order to keep its orientation during the entire range of motion. Note that the tilted parallelogram would result in a vertical movement of the end-effector. To reduce this undesired vertical movement to the minimum, the parallelogram was inclined backwards in the opposite direction by an angle γ (Figure 6). This angle γ will be subject to the optimization of the design parameters after the derivation of the kinematics.

The kinematics of the robot end-effector were derived using homogeneous transformation matrices TBA, representing frame {*B*} in frame {*A*}. The frame {*MCP*} is attached to the MCP joint of the index finger, while frame {*EE*} is attached to the end-effector. The short forms *D*_*x*_, *D*_*y*_, *D*_*z*_ and *R*_*x*_, *R*_*y*_, *R*_*z*_ denote a local translation or rotation respectively (Craig, 2005). The kinematic chain is schematically represented in Figure 6 and mathematically described by Equations (3) to (9).


(3) T0MCP=Dx(x0)Dy(y0)Ry(ψ)Rz(α0)       T10=Rz(α)       T21=Dx(d1)Ry(-γ)Rz(-β0)       T32=Rz(-β)Dx(b)Rz(β)       T43=Rz(β0)Dy(-d2)Ry(γ)Rz(-α0)

(4) T4MCP=T0MCPT10T21T32T43=[x^4y^4z^4p40001]

(5) $$\begin{array}{l} {\hat{x}}_{EE}=\frac{{\hat{y}}_{4}\times {\hat{z}}_{MCP}}{\left\|{\hat{y}}_{4}\times {\hat{z}}_{MCP}\right\|} \end{array}$$

(6) $$\begin{array}{l} {\hat{y}}_{EE}={\hat{z}}_{MCP}\times {\hat{x}}_{EE} \end{array}$$

(7) $$\begin{array}{l} {\hat{z}}_{EE}={\hat{z}}_{MCP} \end{array}$$

(8) $$\begin{array}{l} p_{EE}=p_{4} \end{array}$$

(9) TEEMCP=[x^EEy^EEz^MCPpEE0001]

The matrix T0MCP describes the transformation from the MCP joint frame {*MCP*} to the frame {0}. The local *z*-axis vector ẑ_0_ is coincident with the axis of the principal revolute joint. Actuating this joint by introducing a rotation of α leads to frame {1}. The following transformation T21 places the frame {2} on the first revolute joint of the parallelogram. The next transformation, T32, describes the translation from the input to the output of the parallelogram. The T43 is a transformation that places frame {4} into the end-effector position, whereby ẑ_4_ is always parallel to the principal revolute axis ẑ_0_. The final transformation TEEMCP, which expresses the transformation from the MCP joint to the end-effector, is obtained by constructing ${\hat{x}}_{EE}$ and ŷ_*EE*_ and utilizing the position vector *p*_*EE*_ obtained from Equation (8). The vectors ${\hat{x}}_{EE}$ and ŷ_*EE*_ (Equations 5 and 6) are computed such that ${\hat{x}}_{EE}$ represents the end-effector orientation and are based on the anatomical constraint that the fingertip always moves in the *xy*-plane of the MCP joint frame {*MCP*}. The angle α_0_ + α denotes the angle of the principal axis of rotation, while the angle β_0_ + β is the angular opening of the parallelogram. The angle α describes the rotation introduced by the actuator (α ≥ 0°). The initial angles α_0_ and β_0_ are constant design parameters that need to be optimized. The parallelogram opening angle β was chosen to be proportional to the angle α (with fixed ratio *n*_αβ_, Equation 10) to avoid a too complex mechanical design.


(10) $$\begin{array}{l} \beta =n_{\alpha \beta }\alpha \end{array}$$

**Figure 7:**
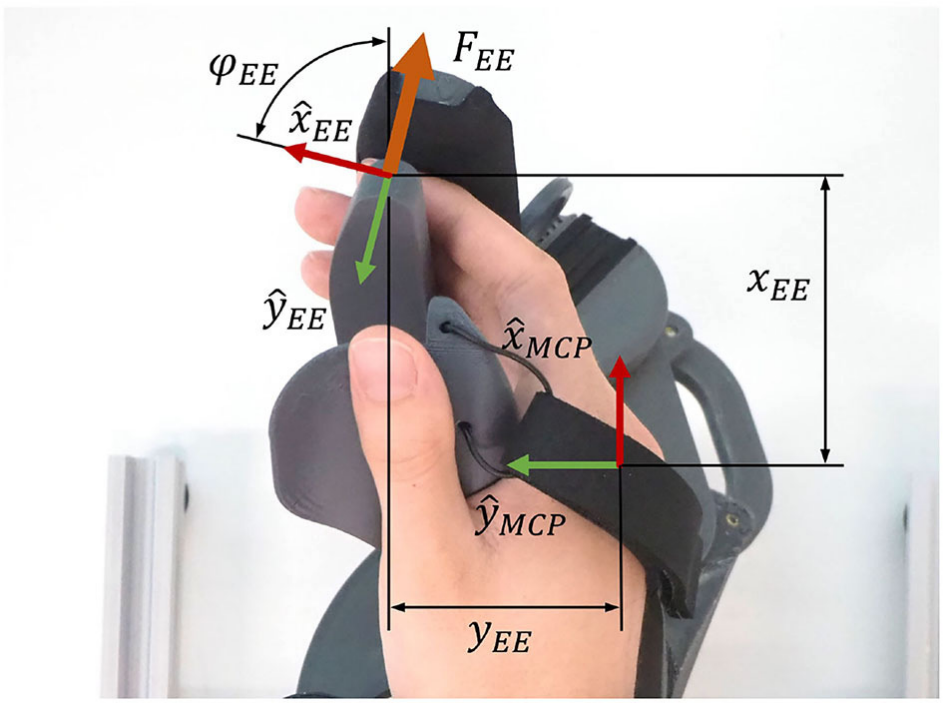
End-effector and MCP joint positions.

The end-effector position coordinates *x*_*EE*_, *y*_*EE*_ and *z*_*EE*_ are obtained from *p*_*EE*_ (Figure 7). The end-effector angle φ_*EE*_, corresponds to the angle between ${\hat{x}}_{EE}$ and ${\hat{x}}_{MCP}$ and is computed by Equation (11). For the upcoming synthesis of the mechanical design parameters, this angle was bounded to $\varphi_{EE}\in \left[-90{}^{\circ},270{}^{\circ}\right)$.


(11) $$\begin{array}{l} \varphi_{EE}=\text{sign}\left(\left({\hat{x}}_{EE}\times {\hat{x}}_{MCP}\right)\cdot {\hat{z}}_{MCP}\right)\text{arccos}\left({\hat{x}}_{EE}\cdot {\hat{x}}_{MCP}\right) \end{array}$$

### 2.3. Optimization of the Design Parameters

Once the the kinematic architecture was decided, we searched for the most suitable set of design parameters by performing an optimization. This optimization step aims to find the optimal position of hands of different sizes on the handle such that their respective fingertip paths overlap. By achieving this, it suffices for the device to track only one common fingertip path, independently of finger sizes. This allowed us to engineer our device without any adjustable moving parts which would have resulted in complicated adjustment mechanisms. Instead, different hand sizes are accommodated by the use of size-specific handles, which can be exchanged within seconds. Four different index finger sizes (*small, small-medium, medium-large, large*) were considered in the optimization, the smallest being the 5th percentile of women and the largest being the 95th percentile of men, according to anthropometric databases. The two intermediate finger sizes were linearly interpolated.

The optimization step required the definition of an adequate cost function whose minimization would result in optimal design parameters. The cost function was defined with two goals in mind:

1. Find interjoint couplings *n*_12_ and *n*_23_ as well as MCP joint positions (*x*_0_, *y*_0_) and initial MCP angles θ_0_ for various hand sizes, such that the overlap of the resulting fingertip paths of different hand sizes is maximized.
2. Find mechanical design parameters (linkage lengths and orientations) such that the robot end-effector closely tracks these overlapping fingertip paths.

In the synthesis of mechanical linkage systems via optimization, the target path is usually known. A common technique is to define the so-called precision points that discretize the target path. These are points along the target path—often at a regular interval—through which the end-effector of the linkage mechanism is supposed to move. In the optimization step, the mechanical design parameters are optimized such that the position difference between the end-effector and these precision points is minimal (Goulet et al., 2016). However, in our case, the target path was not known prior to the optimization. Instead, we needed to simultaneously find the end-effector path as well as a fingertip path for each hand size.

Nevertheless, we had to discretize the still unknown paths. The number of discrete points along the end-effector path was selected to be *n* = 10. A specific point on this path is referred to by the index *j*. The number of hand sizes that were included in the optimization is *m* = 4 and a specific finger size is referred to by *i*. In the upcoming equations, superscripts are used to denote the discretization step *j* and hand size *i*.

To make sure that the fingertip and end-effector paths are optimized along the entire range of motion of the fingers, the optimization variables Δθ^(*i*)^ were introduced. Because the optimal start and end points of the finger paths may slightly differ for each hand size, a hand-size-specific, constant discretization step size was required, defined by Δθ^(*i*)^ > 0. Without this variable, the optimization algorithm might only consider arbitrary, unequally spaced sections of the paths. To allow for a slightly different starting point of the fingertip path of each hand size, the variable $\theta_{0}\in \left[-5{}^{\circ},5{}^{\circ}\right]$ was introduced in Equation (1).

The problem formulation is stated in Equation (12) with the optimization variable vector **x** = [*u, v*, α]^*T*^. The design parameters are constrained to be within the lower bound **x**_*L*_ and the upper bound **x**_*U*_.


(12) $$\begin{array}{l} \underset{x}{\min}\overset{6}{\underset{k=1}{\sum }}f_{k}\left(\text{x}\right)\\ \text{s.t. }{\text{x}}_{L}\leq \text{x}\leq {\text{x}}_{U} \end{array}$$

Each entry in **x** corresponds to a set of design parameters. The mechanical design parameters are included in *v* = [*d*_1_, *d*_2_, *b*, α_0_, β_0_, γ, *n*_αβ_], where each element is a scalar. The rotation around the principal axis α is 1 × *mn*, with *m* denoting the number of considered hand sizes (*m* = 4) and *n* the number of discretized points along the end-effector path (*n* = 10). The design parameters related to the finger kinematics are included in *u* = [*x*_0_, *y*_0_, θ_0_, *n*_12_, *n*_23_, Δθ], i.e., the MCP positions (*x*_0_, *y*_0_), initial MCP joint angles θ_0_, interjoint couplings *n*_12_ and *n*_23_ and Δθ (Figure 4). The upper and lower bounds **x**_*L*_ and **x**_*U*_ were either defined by anatomical constraints (e.g., *n*_12_ ∈ [1.25, 1.75] and *n*_23_ ∈ [0.65, 0.75]) or they were defined such that a reasonable search space for the global optimization was achieved that excludes mechanically infeasible solutions (e.g., very long linkage lengths).

The angle $\theta_{1}^{\left(i,j\right)}$ which is needed to compute the fingertip coordinates $x_{F}^{\left(i,j\right)}$ and $y_{F}^{\left(i,j\right)}$ as well as $\varphi_{F}^{\left(i,j\right)}$ using Equation (1) is computed in Equation (13). All elements in *u* are 1 × *m*, except θ_0_, which is 1 × (*m* − 1) (the initial MCP joint angle θ_0_ of the smallest finger is 0 to avoid redundant optimization variables).


(13) $$\begin{array}{l} \theta_{1}^{\left(i,j\right)}=j\Delta \theta^{\left(i\right)}+\theta_{0}^{\left(i\right)} \end{array}$$

The cost function in Equation (12) consists of a weighted sum of six individual cost functions, each contributing to a specific meaningful goal. Each individual cost function is weighted by specific weights *w*_φ_, *w*_*z*_, *w*_φ, *end*_, *w*_*end*_, *w*_*start*_, and *w*_η_.

- *f*_1_(*x*): This is the fundamental cost function that drives the end-effector path (*x*_*EE*_, *y*_*EE*_, φ_*EE*_) to overlay with the fingertip paths (*x*_*F*_, *y*_*F*_, φ_*F*_) for each point in the path (*j* ∈ {1, *n*}) and each hand size (*i* ∈ {1, *m*}).
  (14) $$\begin{array}{l} f_{1}\left(x\right)=\frac{1}{mn}\overset{m}{\underset{i=1}{\sum }}\overset{n}{\underset{j=1}{\sum }}{\left(x_{EE}^{\left(i,j\right)}-x_{F}^{\left(i,j\right)}\right)}^{2}+{\left(y_{EE}^{\left(i,j\right)}-y_{F}^{\left(i,j\right)}\right)}^{2}+w_{\varphi }{\left(\varphi_{EE}^{\left(i,j\right)}-\varphi_{F}^{\left(i,j\right)}\right)}^{2} \end{array}$$
- *f*_2_(*x*): This individual cost function ensures that there is minimal variation in the *z*-direction (*z*_*EE*_) along the end-effector entire movement.
  (15) $$\begin{array}{l} f_{2}\left(x\right)=w_{z}\frac{1}{m\left(n-1\right)}\overset{m}{\underset{i=1}{\sum }}\overset{n-1}{\underset{j=1}{\sum }}{\left(z_{EE}^{\left(i,j+1\right)}-z_{EE}^{\left(i,j\right)}\right)}^{2} \end{array}$$
- *f*_3_(*x*): This ensures that the angles at the last point (*j* = *n*) of the fingertip path (φ_*F*_) and the end-effector path (φ_*EE*_) are close to φ_*end*_ = 180°.
  (16) $$\begin{array}{l} f_{3}\left(x\right)=w_{\varphi ,end}\frac{1}{m}\overset{m}{\underset{i=1}{\sum }}{\left(\varphi_{EE}^{\left(i,n\right)}-\varphi_{end}\right)}^{2}+{\left(\varphi_{F}^{\left(i,n\right)}-\varphi_{end}\right)}^{2} \end{array}$$
- *f*_4_(*x*): This individual cost function enforces that the last position (*j* = *n*) of the finger paths (*x*_*F*_, *y*_*F*_) from different hand sizes (*i* ∈ {1, *m*}) coincide when the fingers are fully flexed.
  (17) $$\begin{array}{l} f_{4}\left(x\right)=w_{end}\frac{1}{1-m}\overset{m-1}{\underset{i=1}{\sum }}{\left(x_{F}^{\left(i+1,n\right)}-x_{F}^{\left(i,n\right)}\right)}^{2}+{\left(y_{F}^{\left(i+1,n\right)}-y_{F}^{\left(i,n\right)}\right)}^{2} \end{array}$$
- *f*_5_(*x*): This individual cost function reinforces that the robot end-effector and each corresponding fingertip position coincide at the initial point of the paths (*j* = 1). Without this term, the differences between the start of the fingertip paths and the start of the end-effector path tend to be rather large in the *x*-direction. This is especially undesired because in full extension (quasi-aligned in *x*-direction, depending on θ_0_), the fingers are in a singular configuration (or quasi-singular, depending on θ_0_) with respect to the *x*-direction.
  (18) $$\begin{array}{l} f_{5}\left(x\right)=w_{start}\frac{1}{m}\overset{m}{\underset{i=1}{\sum }}{\left(x_{EE}^{\left(i,1\right)}-x_{F}^{\left(i,1\right)}\right)}^{2}+{\left(y_{EE}^{\left(i,1\right)}-y_{F}^{\left(i,1\right)}\right)}^{2} \end{array}$$
- *f*_6_(*x*): This term minimizes the difference between the largest and smallest mechanical advantage along the range of motion. The mechanical advantage η of the mechanism describes the relation between the input torque at the principal revolute axis τ_α_ and the end-effector force orthogonal to the fingertip *F*_*EE*_ (see Figure 7). It is computed by using the Jacobian in the end-effector frame JEE (Equation 19) and varies as a function of the rotation α of the principal axis. Note that the end-effector force *F*_*EE*_ is always pointing in *y*-direction of the {*EE*} frame (see Figure 6). Therefore, the *y*-component of JEE is extracted in Equation (20) to compute the mechanical advantage.
  (19) JEE=TEEMCPT∂pEE∂α
  (20) η(α)=∂yEE∂α=FEEτα=-1[010]JEE
  The addition of this individual cost function is important as variations in the mechanical advantage of a mechanism modify its dynamic behavior and, consequently, its haptic rendering capabilities. Therefore, we want to minimize variations of the mechanical advantage to promote consistent haptic rendering along the entire range of motion of the device.
  (21) $$\begin{array}{l} f_{6}\left(x\right)=w_{\eta }{\left(\text{max}\left(\eta \right)-\text{min}\left(\eta \right)\right)}^{2} \end{array}$$

Due to the non-convex nature of the cost function, a global optimization algorithm was required. The differential evolution algorithm was selected, which was first proposed by Storn and Price (2002) and that has already been successfully applied for the synthesis of mechanical linkage systems (Acharyya and Mandal, 2009; Peñuñuri et al., 2011). The implementation of the “best1bin” strategy in the Python package SciPy (Virtanen et al., 2020) was employed with a relative convergence tolerance of 0.005.

### 2.4. Further Transparency Enhancements: Friction and Gravity Compensation

We aimed at developing a device that is transparent by design. Nevertheless, undesired friction, Coriolis, centrifugal, and gravitational forces could still lower the transparency of a haptic device (Hatzfeld and Kern, 2014), which could possibly also limit self-initiated hand movements, especially in patients who suffer from severe hand paresis. To prevent these disturbing torques from hampering our hand device transparency, we modeled, identified, and compensated the friction and gravitational disturbance forces. Because the gravitational forces and friction cannot be distinguished as seen from the motor, they were modeled and identified simultaneously. The Coriolis and centrifugal forces were neglected in the proposed model, as our solution has low inertias and the target operational speed is relatively low—we estimated a required maximum speed of $\dot{\alpha }$ = 500 °/s based on grasping speeds of stroke patients from Lang et al. (2005) and taking into account the varying mechanical advantage of our device.

We **modeled** the viscous and Coulomb friction and gravitational forces, ${\tilde{\tau }}_{dist}$, following Equation (22), with the parameters to be identified *a*_0_, *a*_1_, *a*_2_, and *a*_3_.


(22) $$\begin{array}{l} {\tilde{\tau }}_{dist}=a_{0}\text{sin}\left(\alpha +a_{1}\right)+a_{2}\dot{\alpha }+a_{3}\text{sign}\left(\dot{\alpha }\right) \end{array}$$

To **identify** the model parameters, we employed an empirically tuned PI velocity controller to track trapezoidal velocity profiles with target velocities $\dot{\alpha }$ = 5, 10, 15,… 150 °/s. The start and the end of the constant velocity plateaus were always located at the same positions by adjusting the acceleration phase (i.e., α = 20°, and α = 160°, respectively). The required torques to sustain the constant velocities—i.e., the output of the PI controller—as well as the velocity and position were recorded at 1 kHz. The proposed model in Equation (22) was fitted to the recorded values by means of a least squares optimization using the trust region reflective algorithm in Python (Virtanen et al., 2020).

We **implemented** the disturbance torque compensation using Equation (23), with the sinusoidal term accounting for the gravitational torque, the second term accounting for viscous friction, and the last one for Coulomb friction.


(23) $$\begin{array}{l} \tau_{comp}=a_{0}\text{sin}\left(\alpha +a_{1}\right)+a_{2}{\dot{\alpha }}_{filt}+b_{0}a_{3}\text{tanh}\left(b_{1}{\dot{\alpha }}_{filt}\right) \end{array}$$

Note that the sign function in Equation (22) was replaced by a hyperbolic tangent function in Equation (23) to obtain a smooth transition around zero speed. A new parameter *b*_1_ was included in the hyperbolic tangent such that the output of the function reaches 0.95 at a speed of ± 5°/s. We also added a second parameter (*b*_0_ = 0.6) to slightly reduce the compensation of the Coulomb friction and ensure that the device remains passive (Schabowsky et al., 2010). The velocity ${\dot{\alpha }}_{filt}$ was computed by backwards differentiation of α which was obtained from the encoder and subsequent filtering with a first-order Butterworth low pass filter (cut-off frequency *f*_*c*_ = 16 Hz).

### 2.5. Evaluation of the Haptic Capabilities of PRIDE

A common measure of **transparency** is the human-robot interaction force in free space (i.e., in the absence of any rendered interaction with virtual objects), which should be minimal (Bernstein et al., 2005; Van Dijk et al., 2013; Just et al., 2018). To benchmark the transparency of the device, six right-handed participants (1 female, 5 male, aged 22 to 36 years, with hand sizes: 1 small, 2 small-medium, 3 medium-large and 1 large) without any known hand impairments were asked to perform finger flexion and extension movements with their right hand installed on the device. Ethical review and approval was not required for the study on human participants in accordance with the local legislation and institutional requirements. The participants provided their written informed consent to participate in this study.

The participants, who were naive to the device/task, were asked to open and close their hands repeatedly in a natural and comfortable manner without reaching the mechanical end-stops of the device. Our goal was to evaluate the transparency of the prototype by measuring the interaction forces during these movements with and without gravity and friction compensation. We equipped the device with a handle corresponding to the participants' individual hand sizes. To obtain comparable measurements, a certain movement frequency was imposed. This was achieved by presenting rhythmic auditive cues to the participants. A metronome was used to present cues with 20, 40, and 60 beats per minute (BPM). The participants were instructed that one movement (flexion or extension) should last one beat. To help the participants with the timing—especially in the 20 BPM condition—we introduced two intermediate beats with a different pitch.

For each movement frequency (i.e., 20, 40, and 60 BPM) and condition (i.e., with and without gravity and friction compensation), participants performed 12 flexion and extension movements, which we refer to as one sequence. The interaction force between the fingers and the fingertip support was measured with a force sensor (TAL 221, SparkFun Electronics, USA) and recorded at ≈ 80 Hz (OpenScale with custom firmware, SparkFun Electronics, USA). The root mean square (RMS) of the interaction forces was computed for each sequence of 12 movements. To evaluate the effectiveness of the friction and gravity compensation, the differences in the RMS of the interaction force were evaluated by a two-way repeated measures ANOVA with gravity and friction compensation (on/off) and BMP (20/40/60 BPM) as within-subject factors.

To demonstrate the device's **capability to render** interaction forces with virtual tangible objects, a virtual wall was also implemented and evaluated in a second benchmark experiment with one healthy participant. Virtual walls are usually represented by either a force derived from a linear virtual spring and damper or by a torque derived from a rotational virtual spring and damper in the end-effector space. In the context of grasping, a virtual wall based on a linear spring and damping, orthogonal to the fingertips would be the obvious choice. However, the orientation of the end-effector force of our device *F*_*EE*_ (and opposing fingertip force *F*_*F*_) depends on α. Consequently, to represent a meaningful virtual wall, based on a linear virtual spring and damper, it was needed to linearize the movement of the end-effector along ŷ_*EE*_. The penetration depth of the fingers into the virtual wall should be relatively small, and therefore, the penetration depth Δ*y*_*EE*_ can be computed employing the following linearization Equation (24):


(24) $$\begin{array}{l} \Delta y_{EE}\approx \frac{\partial y_{EE}}{\partial \alpha }\Delta \alpha =\frac{1}{\eta }\Delta \alpha \end{array}$$

where Δα is the penetration depth in the joint space of the primary axis and η is the mechanical advantage. The momentary linear speed of the end-effector ẏ_*EE*_ is computed using Equation (25):


(25) $$\begin{array}{l} {ẏ}_{EE}=\frac{1}{\eta }{\dot{\alpha }}_{filt} \end{array}$$

The interaction force between the fingers and the virtual wall *F*_*wall*_ is then computed with Equation (26) with *K* and *B* being the desired virtual spring and damping values.


(26) $$\begin{array}{l} F_{wall}=\{\begin{array}{cc} K\Delta y_{EE}+B{ẏ}_{EE} & \;\text{if}\Delta y_{EE}>0\\ 0 & \;\text{else} \end{array} \end{array}$$

This force is then transferred to a motor torque τ_*mot*_ using Equation (27). The friction and gravity compensation torque τ_*comp*_ was added in Equation (27) for an accurate rendering of the virtual wall. The term *n*_*mot*,α_ represents a transmission ratio which translates the motor torque to the torque at the principal axis of rotation, i.e., τ_α_ = τ_*mot*_*n*_*mot*, α_.


(27) $$\begin{array}{l} \tau_{mot}=\frac{1}{n_{mot,\alpha }}\frac{1}{\eta }F_{wall}+\tau_{comp} \end{array}$$

To evaluate the haptic capabilities of the device, a virtual wall was rendered at different positions and the stability regions were evaluated by one additional participant (male, age 29, medium-large hand size). For a given virtual damping *B*, the virtual spring constant *K* was manually varied in steps of ±2 N/mm until the maximum value of *K*, which did not introduce any perceivable oscillations, was found. The stability of the wall was judged according to the criteria proposed by Colgate and Brown (Colgate and Brown, 1994). Contrary to the interaction force evaluation of the device transparency, the test person in this second evaluation was familiarized with the device and the notion of virtual wall stability prior to the evaluation.

## 3. Results

### 3.1. Optimized Design Parameters

**Figure 8:**
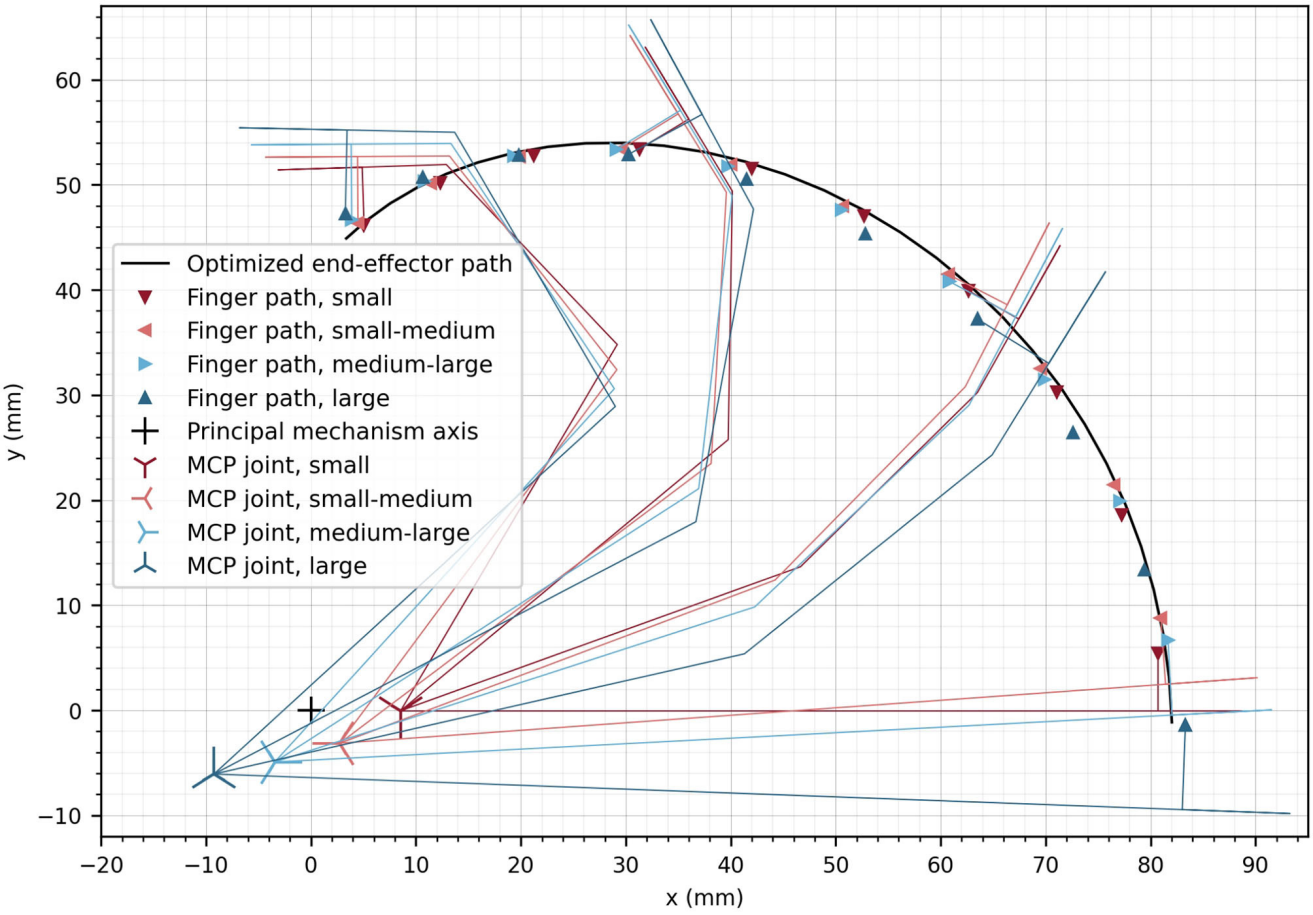
Optimized end-effector path (solid line) and optimized positions of fingers at the discretized finger paths. The optimal location of the MCP joint is displayed for the different hand sizes as crosses. The small cross represents the location of the principle axis of rotation of the device. For a subset of the optimized finger positions, the finger segments are illustrated.

The resulting optimal end-effector path as well as the path of the contact point of the corresponding index finger of each hand size (i.e., four different sizes from small to large) were obtained upon convergence of the differential evolution algorithm after 2425 iterations (Figure 8). Furthermore, the corresponding MCP joint positions, which were found for each hand size, are indicated in Figure 8 with crosses. The deviation of the end-effector path compared to the fingertip paths measured as the mean Euclidean distance between the end-effector points and the corresponding fingertip contact points (for all hand sizes and all discretization steps) remained small (1.14 mm).

**Figure 9:**
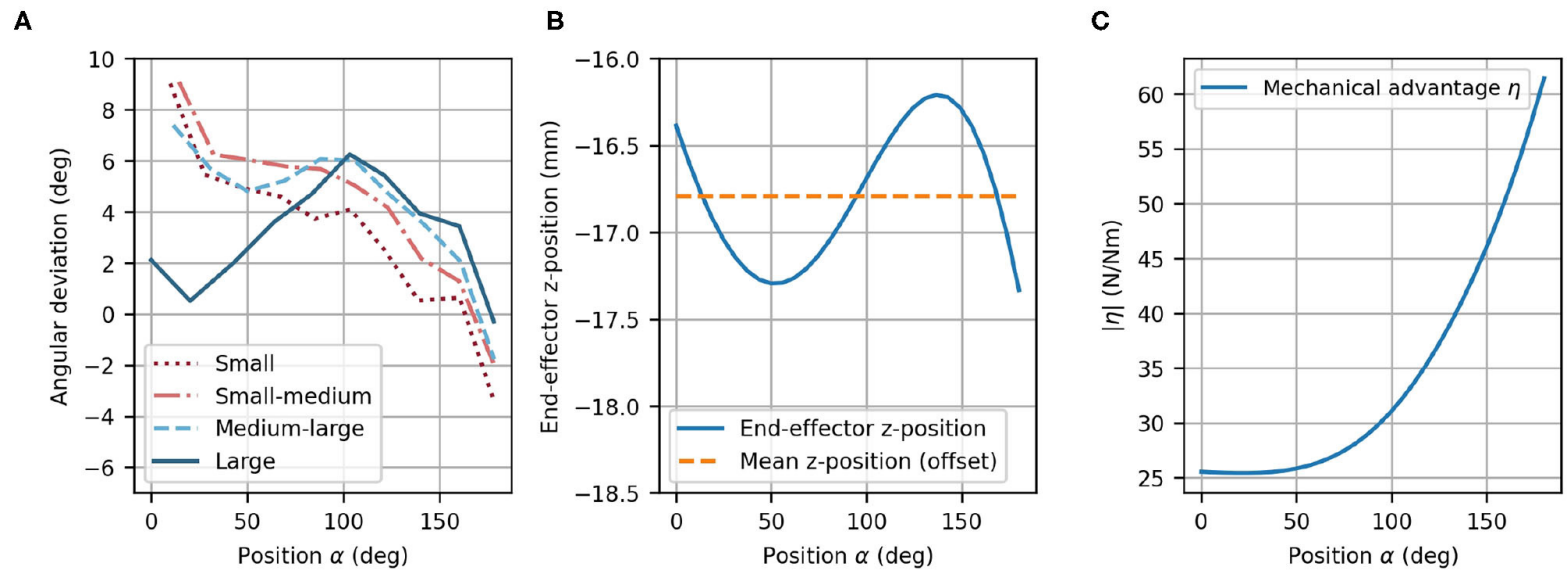
Optimized design parameters. **(A)** Angular deviation of each optimized finger path with respect to the optimized end-effector path as a function of α. **(B)** End-effector *z*-position with offset (dashed line) and variation of the *z*-position (solid line) as a function of α. Note that the offset (mean of *z*-position) was taken into account during the mechanical design. **(C)** The mechanical advantage η increases with α.

The angular deviation of the fingertip angle φ_*F*_ with respect to the device end-effector angle φ_*EE*_ is represented in Figure 9A as a function of α. The mean deviation across finger sizes and full range of motion is only 3.78°. The largest deviations are observed as the finger reach full extension (i.e., ≈ 9° for the two smallest hand sizes for α = 0°). Nevertheless, the observed deviations are within an acceptable range as it would anyway not be possible to drastically constrain the fingertip angle on the device in a comfortable manner. Due to this certain angular compliance from the fingertip fixation, the obtained angular deviations are not noticeable when using the device.

In the optimization, the difference between the fingertip *z*-position and the end-effector *z*-position was not included. Instead, the mean *z*-position of the end-effector can simply be considered during the mechanical design as a constant offset. However, the variation of the *z*-position of the end-effector (in direction of ẑ_*EE*_) was desired to be zero because the fingertips move in the *xy*-plane of {*MCP*}, and has consequently, been minimized in the optimization. The end-effector vertical variation after optimization remains small along the complete end-effector path with a mean deviation of 0.380 mm (Figure 9B). The peak-to-peak vertical variation is less than 1.5 mm, which is not perceptible when using the device.

The mechanical advantage (Figure 9C) depends on α and ranges from η = 25.4 N/Nm to η = 61.4 N/Nm. This results in a change of mechanical advantage along the full range of motion by a factor of 2.42. While η remains low for small values of α, it increases above α ≈ 50°.

### 3.2. Hardware and Mechanical Realization

**Figure 10:**
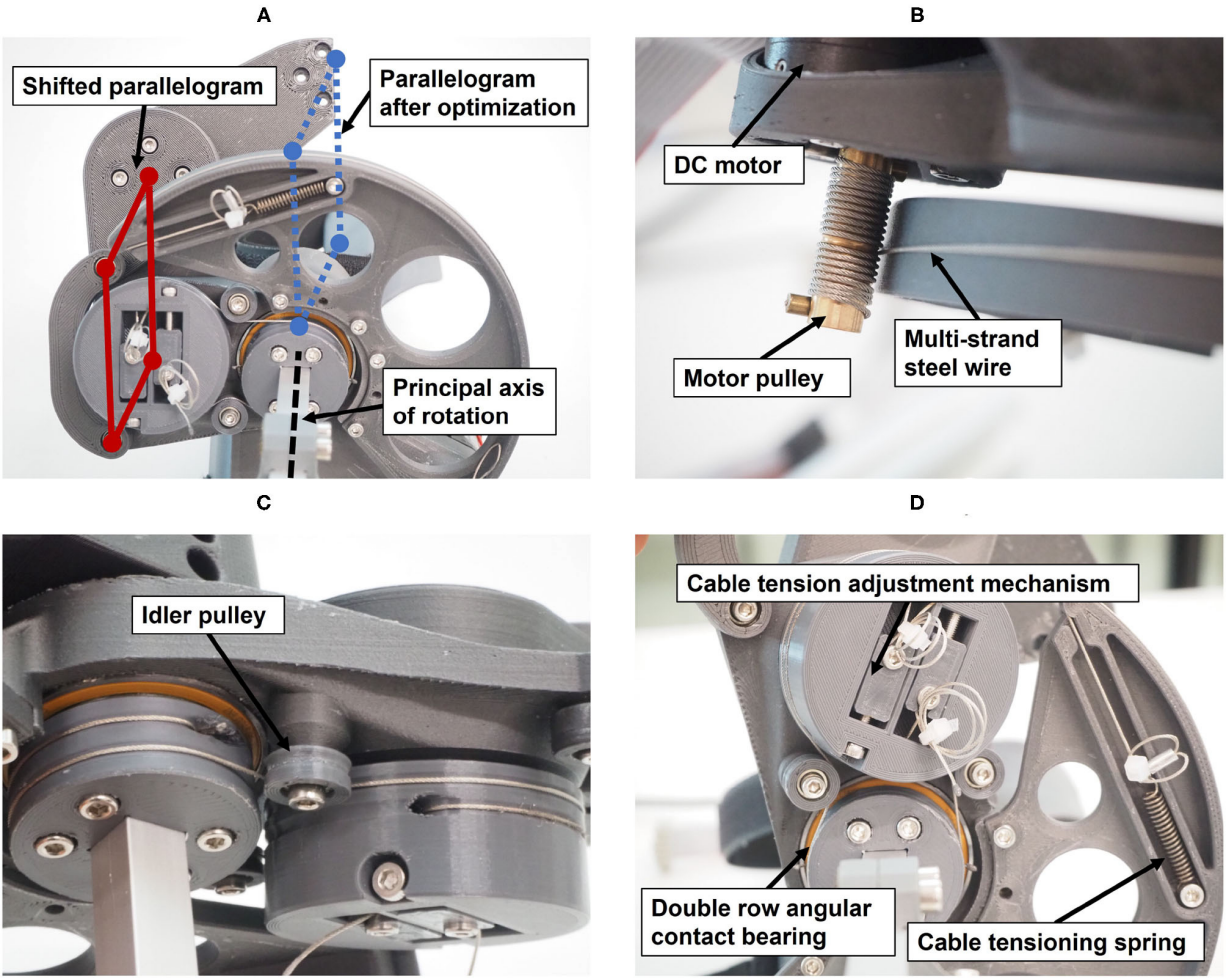
Technical details of the prototype: **(A)** View from below showing the shifted parallelogram. **(B)** Actuation of the principal axis of rotation through a capstan transmission. **(C)** Actuation of the parallelogram, including idler pulley for cable guidance. **(D)** Cable tensioning mechanisms.

The main structure of the resulting robotic hand module design includes a parallelogram with one main arm and a set of bearings, which can be mechanically solicited in any direction. A second light-weight arm with small bearings, which only transmits forces along its longitudinal axis, completes the parallelogram structure (Figure 10A). The parallelogram in the prototype was displaced w.r.t. to its original location within the *xy*-plane of frame {2}. This shift of the parallelogram does not modify the kinematic chain because the corresponding offsets were added between frame {3} and the end-effector frame. However, the shift allows to adapt the mechanical design in order to avoid collisions between the mechanical structure and the user's forearm during finger flexion. Because these offsets would have been redundant in the optimization, they were added during the design of the actual prototype.

**Figure 11:**
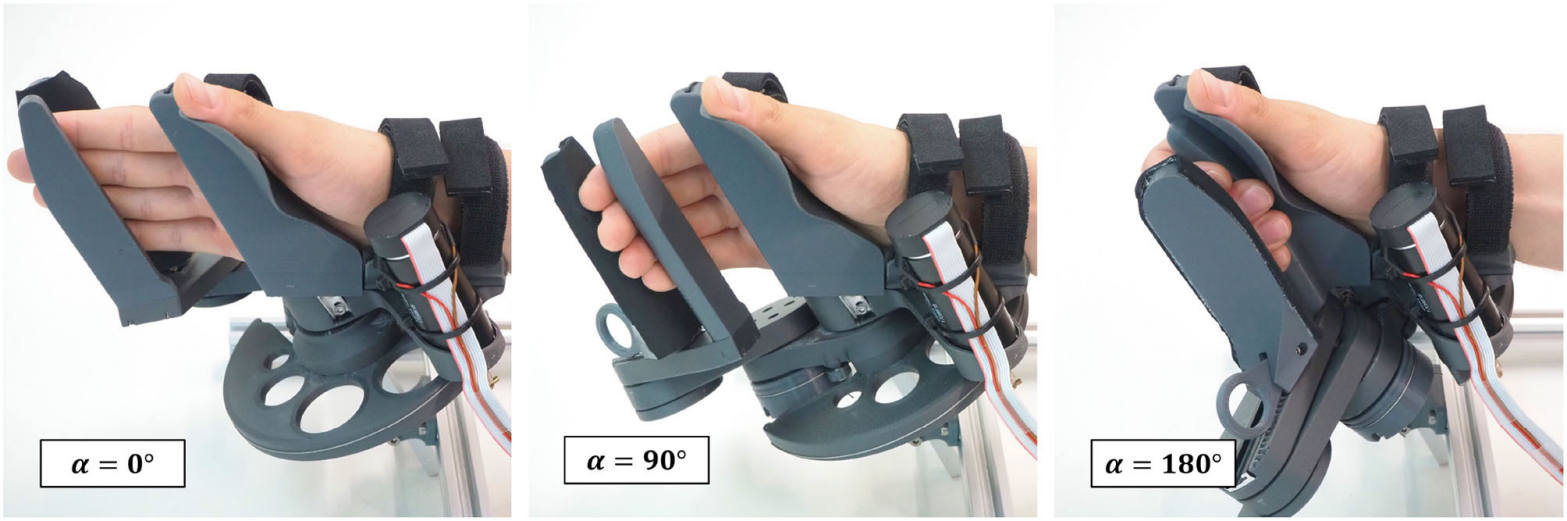
Hand movement sequence from full finger extension to 180° flexion for a hand of size *medium-large*.

**Figure 12:**
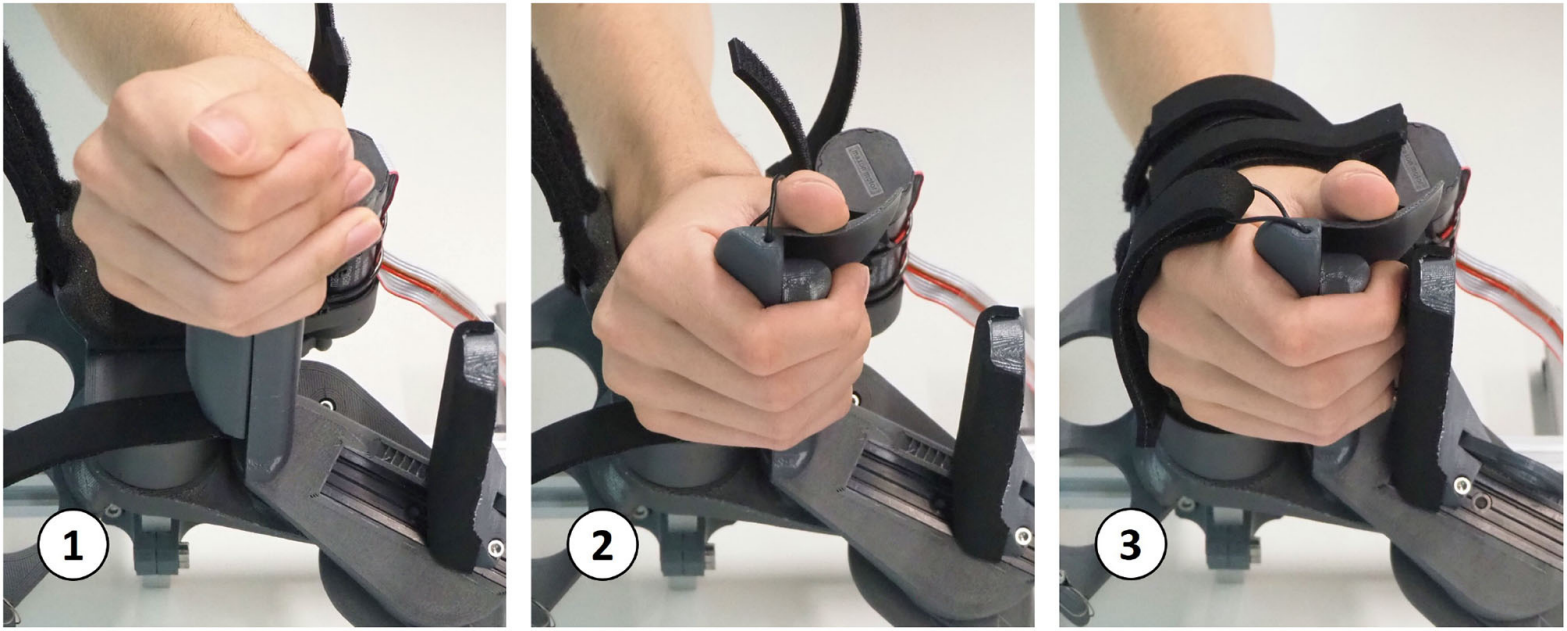
Setup sequence: a hand with flexed fingers can easily be slid onto the handle. Then, within a few seconds, the wrist and hand straps can be tightened and the fingertip fixation can be adjusted.

The PRIDE prototype was manufactured using a combination of 3D printed parts. Carbon-reinforced polylactic acid (PLA) was employed for structural parts and standard PLA for parts that are touched during use (Figure 10). Square aluminium profiles were employed for structural support. For each of the four hand sizes, a specific handle was designed such that it locates a hand of the corresponding size according to the size-specific MCP joint offsets *x*_0_ and *y*_0_, which were obtained from the optimization. To design ergonomic handles and to consider the different depths and breaths of fingers for different hand sizes, anthropometric data from Vergara et al. (2018) was consulted. In each handle, we integrated a cushioned strap which allows to attach the metacarpal bones of the hand. To constrain wrist movements, a wrist rest with two cushioned straps was designed. Finally, to allow the fingers to execute extension movements (Figure 11), a fingertip fixation with a quick-release mechanism was added on the dorsal side of the fingers. All of these fixations were designed to promote a fast and effortless setup as demonstrated in Figure 12.

The actuation of the principal axis is performed by a capstan transmission to reduce the needed motor torque (Figure 10B). A capstan transmission satisfies all listed mechanical requirements in terms of transparency, i.e., it is backlash-free, highly backdrivable, and has low friction. A drive pulley with 7 mm diameter, actuated by a brushed DC motor (RE30, Maxon Motor AG, Switzerland) that includes an encoder with 1,000 ticks per revolution (4,000 in quadrature) drives a large output drum (see Figure 10). The requirement on the minimum force on the fingertips (*F*_*F,min*_ = 30 N) and the minimal mechanical advantage resulted in a output drum of diameter 156 mm. The motor is driven by a custom controller board based on the ones employed in the Sigma.7 haptic device (Force Dimension, Switzerland) and controlled with an update rate of 1 kHz from a PC with a Linux operating system.

The parallelogram is designed to move with a constant mechanical coupling *n*_αβ_ w.r.t to the position of the principal axis α. To retain the low-backlash and high backdrivability of the cable actuation, this coupling was also designed using a cable transmission. The shifting of the parallelogram (Figure 10A) causes the principal axis and the revolute axes of the parallelogram to be skew. A cable transmission is, therefore, also a good solution to easily couple these skew axes of rotation. Small idler pulleys were used to guide the cables from a stationary pulley in frame {0} (whose axis is aligned with ẑ_0_) to a pulley driving the parallelogram. They were arranged so that they need minimal space (see Figures 10C,D).

### 3.3. Transparency and Virtual Wall Tests

**Figure 13:**
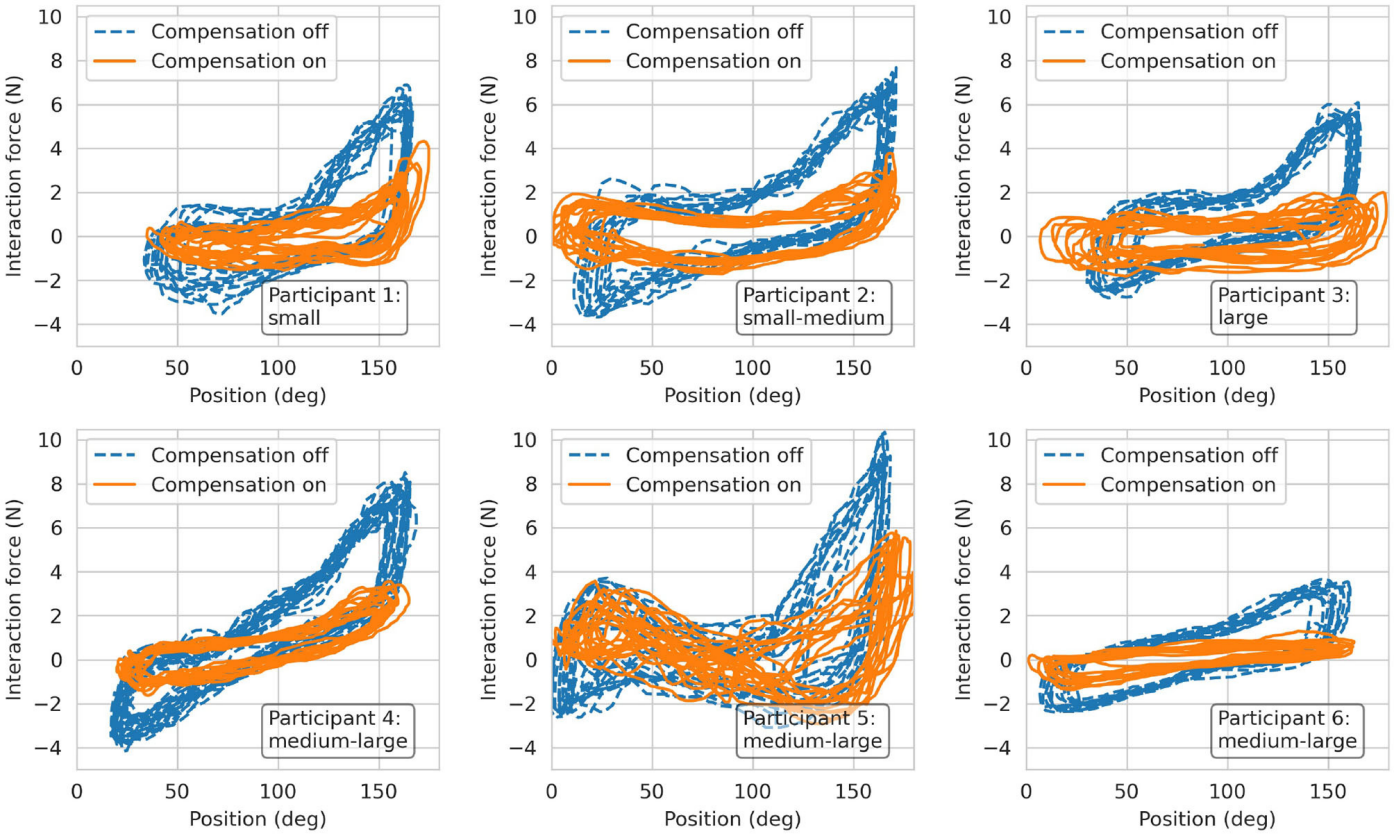
Results from the transparency test. Interaction forces recorded from the six participants during finger flexion and extension movements at 60 BPM.

The transparency test revealed a RMS human-robot interaction force of 2.96 N before the compensation of disturbance forces. The addition of gravity and friction compensation reduced this value to 1.37 N for all movement frequencies—i.e., BPM—combined (Table 1). The results of the repeated measures ANOVA revealed a statistically significant main effect of the compensation (*p* < 0.001) and a non-significant effect of BPM (*p* = 0.055) as well as interaction effect (*p* = 0.094). Hence, the effectiveness of the gravity and friction compensation to enhance transparency was confirmed. Figure 13 shows the interaction forces for each participant as a function of the end-effector position α.

**Table 1:** RMS of human-robot interaction forces (*N*) during continuous finger flexion and extension movements at different frequencies.

**Compensation**	**BPM**			
	**20**	**40**	**60**	**Combined**
Off	3.02 (0.52)	2.90 (0.64)	2.96 (0.71)	2.96 (0.62)
On	1.55 (0.49)	1.36 (0.48)	1.20 (0.47)	1.37 (0.50)

*The values inside the brackets denote the standard deviation*.

**Figure 14:**
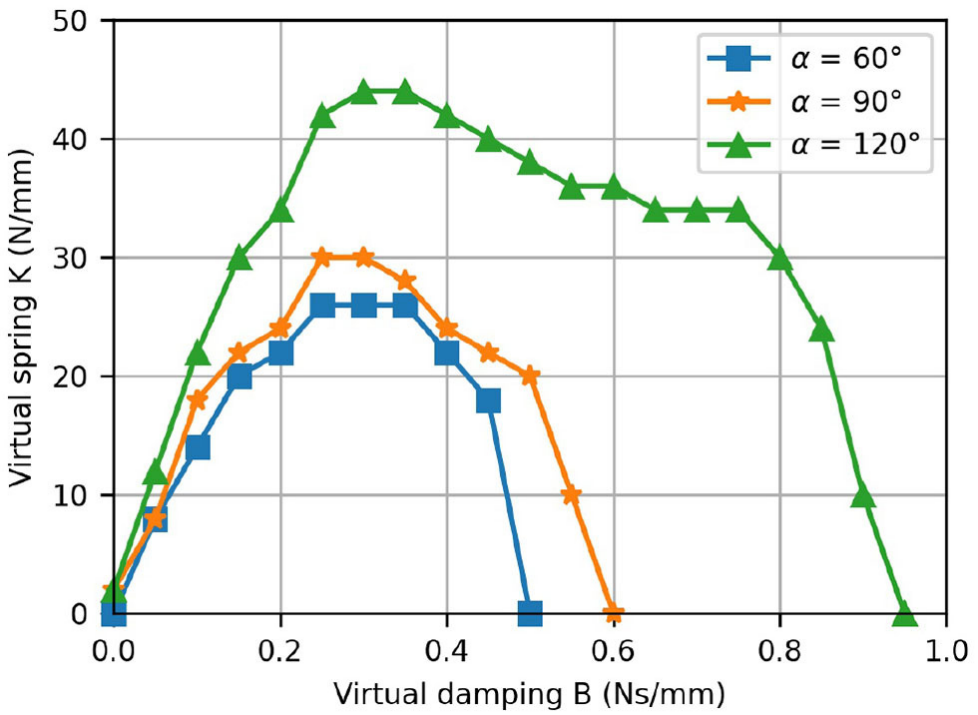
Results from the virtual wall test. The K-B plot shows the stability regions for the rendering of a virtual wall located at different positions.

The stability regions—i.e., the areas beneath the plotted points—resulting from the interaction with virtual walls at different positions (α = 60°, 90° and 120°) are depicted in Figure 14. Note that as the wall position increases—i.e., for larger α—the stable region becomes larger.

## 4. Discussion

In this article, we present PRIDE (Palmar RehabilitatIon DEvice), a novel device for sensorimotor hand rehabilitation based on clinical and anatomical requirements gathered from interviews and questionnaires with clinical personnel. Clinicians reported that special attention should be paid to high usability and effortless setup and that the device should support the practice of finger flexion/extension (Rätz et al., 2021). This is in agreement with the findings of Lang et al., who report that poor grasping performance post-stroke is related to a lack of adequate finger extension ability (Lang et al., 2009). Further requirements were established based on anatomical considerations—e.g., different lengths of the individual fingers within a hand—as well as ergonomic aspects in cylindrical grasping. The need for a device with high-quality haptic rendering capabilities to provide sensorimotor neurorehabilitation added further mechanical requirements, e.g., high backdrivability, low backlash, and high transparency.

A novel kinematic design based on a novel architecture with only one actuator was developed to meet the gathered clinical, anatomical, and design requirements. To accommodate patients with different hand sizes, our device accurately tracks and supports the fingertip paths of hands of various sizes. To achieve this, we performed an optimization based on anthropometric data from hands of different sizes and anatomical considerations (e.g., constant interjoint coupling) to determine the device mechanical design parameters. In a feasibility test with seven healthy young participants with different hand sizes, the specific functionalities of the device were demonstrated, namely, physiological finger movements with large range of motion, quick setup, high transparency, and fine haptic rendering. In the following subsections, we discuss the novelties of our hand rehabilitation haptic device, the study limitations, and future work.

### 4.1. Our Palmar Device Allows for an Effortless Installation of the Patient's Hand While Offering Large Physiological Finger Flexion/Extension Motions

The rapidly growing number of published works on hand rehabilitation shows that a great number of hand rehabilitation devices have been developed for research and commercial purposes [see our comparison table in the Supplementary Material and (Bos et al., 2016; Gassert and Dietz, 2018)]. Our literature research on hand rehabilitation devices resulted in a total of 54 devices, both commercial [e.g., Amadeo^®^ (TyroMotion, Austria), Manovo^®^ Power (Hocoma, Switzerland)] or research prototypes (e.g., Taheri et al., 2014; Zhu et al., 2014; Cheng et al., 2018). Nevertheless, only a few of these devices were developed with strong focus on high usability and easy setup (e.g., Masia et al., 2007; Yap et al., 2016; Randazzo et al., 2018; Bützer et al., 2020). While hand exoskeletons are inherently difficult to setup in patients suffering from spasticity due to their complexity (Aggogeri et al., 2019), grounded end-effector devices generally compromise the range of finger motion (e.g., Masia et al., 2007; Zhu et al., 2014; Just et al., 2019) and/or are not able to guarantee physiological movements of the fingers (e.g., Dovat et al., 2008; Hioki et al., 2011; Metzger et al., 2011).

To maximize the clinical applicability and acceptance of our novel device, we collaborated closely with clinical personnel. We began our development by conducting a survey with 33 healthcare professionals while we continuously integrated feedback from therapists in several prototype iterations. The repeatedly mentioned need for an effortless and rapid patient setup from our clinical partners, led us to the development of a palmar device with a compact handle geometry. This allows to install even a clenched hand on the device by sliding it onto the handle, similar to Just et al. (2019). The reported need for intensive training of finger extension movements motivated us to develop a design that supports finger movements from full flexion up to full extension. Thus, in a minimally actuated device, we could fulfill two important clinical requirements: a quick effortless setup and training of physiological full finger flexion/extension movements.

### 4.2. The Kinematic Solution Ensures That All Fingers Are Supported Through the Full Range of Motion and That the Little Finger Does Not Lose Contact in Finger Extension

To accommodate for the different lengths of the individual fingers of a given hand, the contact points of the fingers with the robot end-effector were defined to lie on a line which was rotated by an angle ψ = 25° relative to the transverse axis of the hand. This was especially important to not loose contact between the little finger and the robot end-effector during finger extension due to its generally shorter length w.r.t. the other fingers. Keeping the orientation of the longitudinal axis of the end-effector invariant during the entire range of finger motion, resulted in a remarkably natural grasping motion. Our resulting novel kinematic design is—to the best of our knowledge—the first palmar device for sensorimotor hand rehabilitation which supports physiological movements for index, middle, ring and little finger over a range of motion as large as $\varphi_{F}\in \left[0{}^{\circ},180{}^{\circ}\right]$.

### 4.3. Our Design Allows for a Natural Cylindrical Grasp

We took into consideration that cylindrical objects are usually grasped such that the longitudinal axis of the object runs obliquely from the MCP joint of the index finger to the base of the hypothenar eminence by rotating the cylindrical handle forwards by ψ = 25° w.r.t to ẑ_*MCP*_. In combination with the aforementioned invariant orientation of the longitudinal axis of the end-effector, we ensured that the fingertip of the little finger is more proximal than the fingertip of the index finger through the entire range of motion (see Figure 11) which results in a natural, functional cylindrical grasp. To better understand this, it should be noted that the MCP joint of the little finger is generally located in a more proximal and more palmar position than the MCP of the index finger. Further, Hayashi et al., report a higher functional range of motion of the MCP joints of ring and little finger compared to index and middle finger during power grasps (Hayashi and Shimizu, 2013). These findings may explain why the fingertip of the little finger is not only more proximal in full extension—as one might expect due to the shorter length—but along the full motion of a cylindrical grasp up to full flexion.

Yet, even though we achieved a natural cylindrical grasp by tilting the handle and the end-effector, it comes with a drawback: Apart from full finger extension or flexion, the effective finger force applied at the end-effector also has a vertical component along ẑ_*EE*_, which is not considered in *F*_*F*_—i.e., the finger force *F*_*F*_ is applied with a certain, position-dependent angle (maximum value of 25° at α = 90°) relative to the end-effector. This could potentially result in slipping or sensations of tangential forces on the fingertips.

### 4.4. The Device Guarantees Physiologically Correct Finger Movements for a Large Variety of Hand Sizes

Due to the achieved large range of motion, our device needed some kind of adjustment for different hand sizes. This is contrary to other palmar devices with a smaller range of motion like the handle of Just et al. (2019) or Alpha-Prototype II (Masia et al., 2007), which do not require any adjustments for different finger sizes (see comparison table in Supplementary Material). Nonetheless, based on the clinicians' feedback, we aimed to avoid any adjustments of the moving parts of the device because they would have resulted in a drastical increase of complexity and long setup times (e.g., Schabowsky et al., 2010; Cheng et al., 2018; Marconi et al., 2019). Instead, we engineered several handles—that can be exchanged within a few seconds—to position hands of different sizes such that their respective fingertip paths overlap. These engineered handles allow to position the MCP joints of different hand sizes in optimal locations to grant the device end-effector to track only one common fingertip path, independently of the hand size. To achieve this goal, constant interjoint couplings were considered, which, even if it is a simplification of the biomechanical functioning of the hand, results in physiological movements of the fingers. Intermediate meetings with therapists confirmed that exchangeable handles are appreciated and valued as a considerable advantage compared to adjustment of the device itself. Furthermore, the exchangeability facilitates the cleaning and disinfection of the device, which leads again to shorter setup times.

The synthesis of the mechanical design was performed utilizing a differential evolution optimization algorithm (Storn and Price, 2002) due to the non-convex nature of the problem. Four representative hand sizes, from small to large according to anthropometric databases (Garrett, 1970a,b; Buchholz et al., 1992; Vergara et al., 2018), were included. The goal was to simultaneously find mechanical design parameters as well as the common fingertip path for various hand sizes. A cost function, consisting of a set of individually weighted functions, was developed to find suitable design parameters. Having too many weights in a cost function can potentially lead to cumbersome and inefficient trial and error tuning of the weights (Yang, 2014). However, each of the utilized individual functions has an intuitive meaning, which allowed us to define the weights with relative ease. This cost function could also have been partially replaced by non-linear constraints. Yet, it would have been challenging to define and justify the bounds of these constraints.

Using this optimization approach, we found design parameters with a mean Cartesian position and angular difference between the optimized points of the finger paths and the end-effector path of 1.14 mm, respectively 3.77°. The higher angular deviations compared to the Cartesian position deviations are desired and can be explained by the attribution of the weights in the cost function, as an angular deviation of 3° was equally penalized as a 1 mm Cartesian deviation in our defined cost function.

With our chosen kinematic design, a certain vertical movement of the end-effector can not be eliminated completely, but we were able to reduce it enough (peak-to-peak less than 1.5 mm) to not be perceptible nor uncomfortable. It has to be noted though, that an even more appropriate movement of the end-effector could possibly be found by actively including the kinematics of all four fingers in the design optimization. However, this would have needed a reliable database for all MCP, PIP, and DIP joint positions and orientations of different hand sizes, as the joint axes of the middle, ring, and little fingers are not parallel to the transverse axis of the hand (Kapandji, 1982).

### 4.5. PRIDE Allows for High-Quality Haptic Rendering

The large intrinsic mechanical advantage of our design, in combination with the selected capstan cable transmission, allows to achieve high grasping forces without the need for additional gearing to increase the motor torque. As a result, the mechanical transmission is highly backdrivable and transparent. Even though no force sensors were utilized for the control of our novel device, the RMS of the user-robot interaction forces reached a maximum of only 2.96 N during a highly dynamic task. We further significantly reduced these undesirable interaction forces to a maximum of 1.37 N, by modeling, identifying, and compensating the friction and gravitational disturbance forces. These residual forces are on a similar level as other haptic devices for hand rehabilitation, e.g., Metzger et al. declare interaction forces of 1.5 N for the ReHapticKnob (Metzger et al., 2012), Schabowsky et al. report approximately 2 N (with an assumed finger length of 100 mm) for the HEXORR (Schabowsky et al., 2010), and Endo et al. report an impressive 0.1 N per finger for the HIRO III haptic interface (Endo et al., 2011). Unfortunately, the interaction force has not been systematically reported in any of the other reviewed devices.

The friction compensation could be further ameliorated by improving the speed resolution of our device or by implementing a more sophisticated speed estimation algorithm, e.g., by using first-order adaptive windowing (Janabi-Sharifi et al., 2000) or a Kalman filter (Taheri et al., 2014). The gravity compensation torque estimation could also be improved by using a more accurate model instead of the suggested sinusoidal approximation, e.g., by taking into account all moving parts of the kinematic chain. Finally, bearings with less static friction could be used, or a force sensor could be permanently integrated into the handle to perform closed-loop impedance control, at the expense of a more costly solution.

The measured K-B plot demonstrated the excellent haptic rendering capabilities of our device as the achieved stability regions are comparable to other devices which were specifically built for high-fidelity haptic hand rehabilitation. For the ReHapticKnob, maximum values of *K*= 50 N/mm, respectively *B*= 0.25 Ns/mm were reported (Metzger et al., 2012). The ETH Mike achieves values of *K*= 8 N/mm and *B*= 0.04 Ns/mm (assuming a lever-arm of 100 mm). In Figure 14, it is apparent that the stability region depends on the position of the virtual wall and increases with the position. This can be explained by the varying mechanical advantage, which has two effects. First, it modifies the device inertias and, especially, the reflected inertia of the motor. Second, it also inherently improves the speed resolution as the fingers are more flexed. That said, the fundamental drawbacks of K-B plots should also be noted. The stability of a virtual wall depends on the user interaction—i.e., the admittance of the user's hand. Besides, even though Colgate and Brown provide a definition for the stability of a virtual wall, the wall might be perceived slightly different across different users (Colgate and Brown, 1994).

### 4.6. Study Limitations

Our design procedure suffers from some limitations. First, we did not investigate how more complex interjoint relations (e.g., polynomial) would have affected the mechanical design of the device. By altering the spiral-like movement of the fingertips, this could potentially have resulted in a different end-effector path with lower variation of the mechanical advantage. Furthermore, for our standardized handle sizes, we assumed hand breadths and finger thicknesses to scale proportionally to hand lengths. This might result in inadequate handles, and therefore, suboptimal hand positioning for certain hands. Finally, the choice of 3D printing as manufacturing process, although enabling us to iterate numerous times after feedback from therapists, might have limited the rigidity of the device due to the characteristics of the employed 3D printing materials. Albeit no deflection is visible during normal usage, the (potentially) low stiffness of our 3D printed parts could result in an inaccurate representation of the virtual wall rigidity *K* because any deflection of the end-effector caused by deformations of the parts cannot be sensed by the motor encoder.

The device benchmarking also suffers from a couple of limitations. First, for the performed robot-user interaction force measurements, we assumed that all interactions forces occur orthogonal to the longitudinal direction of the fingertip in the *xy*-plane of the end-effector frame. However, especially if the participant's hand was flexed, it is likely that also tangential forces in the direction of ${\hat{x}}_{EE}$ were transmitted to the end-effector. This could have influenced the force measurements and might explain the observed between-subjects variance, especially in hand-flexed poses. Secondly, the sample size in the interaction force evaluation was rather small. However, given the highly significant main effect observed in the two-way repeated measures ANOVA, it is unlikely that larger sample sizes and/or any other statistical test (e.g., non-parametric) would have shown a non-significant effect of the friction compensation.

Finally, it is currently possible—for a healthy participant—to actively pull the fingertips out of the fingertip fixation. Although it did not occur by accident during the experiments, participants pointed out that this could be a design limitation. The fingertips are constraint by the palmar end-effector contact surface and the dorsal quick-release finger fixation. This allows for an optimal transmission of the forces that occur during a grasp, but does only slightly restrict movements along the longitudinal fingertip axis. Currently, the cushioning of the dorsal fixation does not account for different diameters of the individual fingers, which results in non-uniform pressure on the fingertips and consequently a suboptimal fingertip fixation.

### 4.7. Future Work

While our prototype is functional, it could be further improved with future work. The compact handle geometry introduced a challenging aspect to the design of the handle: Without a thumb rest, the thumb could easily collide with the fingertips of the fingers attached to the end-effector. This is particularly problematic if the thumb is flexed while the end-effector is in extension. During the movement of the end-effector back to flexion, the thumb could get pinched, which must be strictly prevented. The integration of a thumb rest, which prevents this issue, makes the design of the different handles notably challenging. In future developments, this thumb rest should be addressed and improved. Ideally, the device should be extended by an active or passive mechanism which allows the thumb to move into opposition and/or flexion to practice precision grasp. At the same time, the dorsal fixation of the fingertips could be further ameliorated to rule out any involuntary release of the fingertips, i.e., by extending it to the proximal direction and by improving the cushioning. Future work also includes the development of a solution for training the left hand. A mirrored version will be built to allow for training left and right hands or to perform bi-manual tasks.

Finally, the novel device was designed with high usability and low setup times in mind. Although preliminary tests with healthy participants confirmed that the setup is quick and only takes a few seconds, its usability needs to be evaluated with brain-injured patients with different spasticity levels. In future experiments with brain-injured patients, we will further evaluate the clinical practicability of the design.

## 5. Conclusion

In this paper, we presented a novel robotic device for sensorimotor hand rehabilitation. The design is strongly motivated by a set of clinical, anatomical, and mechanical requirements that we established prior to the development. After carrying out the design synthesis via an optimization approach, a functional prototype was built and its haptic capabilities demonstrated in a preliminary test with seven participants. With the clinical-driven design, our robotic hand rehabilitation device has the potential to enable sensorimotor hand rehabilitation for patients with various levels of hand impairment. Moreover, we hope that our design approach will raise the awareness of clinical acceptance and applicability in future research and development of hand rehabilitation devices.

## Data Availability Statement

The datasets presented in this study can be found in online repositories. The names of the repository/repositories and accession number(s) can be found below: https://zenodo.org/record/5542327.

## Ethics Statement

Ethical review and approval was not required for the study on human participants in accordance with the local legislation and institutional requirements. The patients/participants provided their written informed consent to participate in this study.

## Author Contributions

RR and LM-C developed the kinematics as well as the mechanical components of the device. RR and RM established the requirements and conducted meetings with therapists from the Department of Neurology, University Hospital Bern (Inselspital), Switzerland to discuss intermediate prototypes. FC contributed to the development of the motor control electronics and firmware. RR and LM-C edited and revised the manuscript. All authors contributed to the article and approved the submitted version.

## Funding

The present research was supported by the Innosuisse grant 32213.1 IP-CT Novel Clinical-Driven Robot-Assisted Sensorimotor Therapy in collaboration with Force Dimension (Switzerland). The funder was not involved in the study design, collection, analysis, interpretation of data, the writing of this article, or the decision to submit it for publication. This work was also supported by the Swiss National Science Foundation through the Grant PP00P2163800.

## Conflict of Interest

FC is currently employed by Force Dimension (Switzerland), which provided the motors and drivers for the mechanical realization of the prototype as part of their engagement as industrial partner in the Innosuisse project 32213.1 IP-CT “Novel Clinical-Driven Robot-Assisted Sensorimotor Therapy.” The involvement of FC was limited to the development of the prototype and verification of the technical correctness of the manuscript. Our work does neither evaluate, nor promote any commercial devices of Force Dimension. The remaining authors declare that the research was conducted in the absence of any commercial or financial relationships that could be construed as a potential conflict of interest.

## Publisher's Note

All claims expressed in this article are solely those of the authors and do not necessarily represent those of their affiliated organizations, or those of the publisher, the editors and the reviewers. Any product that may be evaluated in this article, or claim that may be made by its manufacturer, is not guaranteed or endorsed by the publisher.
