# Supplementary material for: A Novel Clinical-Driven Design for Robotic Hand Rehabilitation: Combining Sensory Training, Effortless Setup, and Large Range of Motion in a Palmar Device
Source: Front Neurorobot. 2021 Dec 20;15:748196. doi: 10.3389/fnbot.2021.748196 (PMC8721892; doi:10.3389/fnbot.2021.748196)
Supplement: Supplementary file 1 [file Data_Sheet_1.pdf]

Supplementary Material

1 COMPARISON TABLE

Table S1 contains the results of our literature research on robotic hand rehabilitation devices. We restricted our research to devices that allow to train grasping movements and did not include any devices for general upper-limb training. If one of the evaluation criteria was not mentioned in the cited publication or if the device does not possess the functionality described by the evaluation criterion, we marked it with a “-” in the corresponding table entry.

In the **Description**, a very brief summary of the device characteristics is given. It is heavily based on the features which are highlighted by the authors in the corresponding publication. The **DoF** reports the measured and actuated degrees of freedom, the **RoM** describes the range of motion and the **Force** states the achieved continuous force of the devices. In **Setup & adjustments**, we report the necessary adjustments for different hand sizes. This is based on the information available in literature and is not an exhaustive description of the setup process. Finally, in **Haptic rendering**, we list the haptic rendering capabilities of the devices. If available, we present the maximum achieved stable K and B values (i.e., the virtual stiffness and damping of a virtual wall). Note that these values might not occur simultaneously. We refer the interested reader to the corresponding publication for further details.

Table S1: Comparison of robotic devices for hand rehabilitation

**Device**	**Description**	**DoF**[1]	**RoM**[2] **(°)**	**Force (N)**	**Setup & adjustments**	**Haptic rendering**
(Decker and Kim, 2017)	Underactuated exoskeleton with inner glove	12 / 5	0-62 (MCP), 0-88 (PIP)	14	Adjustable rubber straps	yes, with additional vibrotactile stimulation
(Rudd et al., 2019)	Low-cost soft robotic glove, tendon-driven	5 / 5	-	9	Customized parts required for each hand size	-
Reha-Digit, (Hesse et al., 2008)	Passive finger mobilization by actuated plastic rolls	1 / 1	-	-	One size fits all	-
HX-β, (Marconi et al., 2019)	Cable-driven index finger-thumb exoskeleton	9 / 5	0-60 (MCP), 0-60 (PIP), 0-60 (DIP)	4	Adjustable lengths of linkages	-
(Yang et al., 2016)	Jointless tendon-driven exoskeleton	1 / 1	0-90 (MCP), 0-90 (PIP), 0-90 (DIP)	1.5	-	-
HWARD, (Takahashi et al., 2005)	Pneumatically actuated hand orthosis with collective actuation of index to little finger, palm is free	3 / 3	25-90 (fingers overall)[3]	-	Adjustable to hand size	-
Amadeo, (TyroMotion, Austria)	End-effector finger trainer with linear actuators	5 / 5	-	-	Adjustable wrist orthosis, magnets are taped to fingertips	no, but haptic sensitivity training using vibrations
BRAVO, (Leonardis et al., 2015)	Hand exoskeleton with intrinsic adaptability to different hand sizes	2 / 2	-	30	Intrinsic adjustment	-
Hand of Hope, (Ho et al., 2011)	EMG-driven exoskeleton	5 / 5	0-55 (MCP), 0-65 (PIP)	-	Adjustable lengths of linkages	-

[1] Measured / acutated degrees of freedom. [2] Range of motion [3] The ranges of motion or forces for some degrees of freedom are missing in the table, see reference for additional information.

Device	Description	DoF[1]	RoM[2] (°)	Force (N)	Setup & adjustments	Haptic rendering
MERLIN / ArmAssist, (Proxima Medical Technology, Serbia)	Holonomic omni-wheel-driven robotic platform with passive grasping mechanism	-	-	-	-	-
CADEX, (Kim and Park, 2018)	Tendon-driven exoglove for dexterous motion	7 / 7	27-65 (MCP), 0-73 (PIP), -13-34 (DIP)[3]	12	Stretchable fabric, one size fits all	-
CHAD, (Alnajjar et al., 2021)	Assistive, tendon-driven soft robotic glove with integrated drive unit on forearm	3 / 3	-	28	Designed for medium-sized arm/hand	-
(Huang et al., 2020)	Tendon-driven glove with bi-directional actuation	4 / 4	0-31 (MCP), 0-35 (PIP), 0-57 (DIP)	2.5 per finger	Designed to fit the author	-
SCRIPT SAO-i3, (Ates et al., 2015)	Actuated hand and wrist orthosis for assisted extension movements	1 / 1	0-150 (fingers overall)[3]	4-8	Based on SaeboFlex which is available in mutliple sizes	-
CyberGrasp, (CyberGlove Systems, USA)	Cable-driven force feedback glove	5 / 5	-	12 per finger	Must be combined with CyberGlove sensorized glove	yes
(Pu et al., 2020)	Cable-driven exoskeleton	5 / 5	-	-	-	-
(Sooraj et al., 2013)	Haptic orthosis for hand and forearm	6 / 6	0-85 (MCP), 0-100 (PIP), 0-80 (DIP)[3]	8-15 per finger	Adjustable to hand size, with self-adjusting parts	yes
ETH-Mike, (Zbytniewska et al., 2019)	Haptic device for proprioceptive and sensorimotor assesment of index finger	1 / 1	0-90 (MCP only)	5.1 Nm (MCP, peak)	Exchangeable handles	yes, K=80 Nm/rad, B=0.4 Nms/rad
Alpha-Prototype II, (Masia et al., 2007)	Haptic handle of MIT-Manus manipulandum	1 / 1	-	70 - 120	One size fits all	yes, K=50 Nm/rad, B=1 Nms/rad

[1] Measured / acutated degrees of freedom. [2] Range of motion [3] The ranges of motion or forces for some degrees of freedom are missing in the table, see reference for additional information.

Device	Description	DoF[1]	RoM[2] (°)	Force (N)	Setup & adjustments	Haptic rendering
ReHapticKnob, (Metzger et al., 2011)	Grounded haptic device for sensorimotor grasp training	2 / 2	30 mm-200 mm (finger flexion)[3]	80	One size fits all	yes, K=50 N/mm, B=0.25 Ns/mm
(Cheng et al., 2018)	Modular cable-driven hand exoskeleton	9 / 9	0-116 (PIP), 0-124 (DIP)	-	Adjustable lengths of linkages	-
(Zhang et al., 2014)	Exoskeleton with circuitous joints	6 / 6	-	7.5 - 11	Adjustable lengths of linkages	-
(Sandoval-Gonzalez et al., 2016)	Exoskeleton with force-position control	14 / 10	-	-	Adjustable lengths of linkages	yes
ReHand, (Wang et al., 2018)	EMG and voice controlled exoskeleton	2 / 2	0-65 (MCP), 0-65 (PIP)	-	Adjustable for different palm sizes	-
(Sarac et al., 2016)	Underactuated exoskeleton with self-adaptability to different finger sizes	3 / 3	0-85 (MCP), 0-100 (PIP), 0-80 (DIP)	1.5 Nm (MCP), 0.4 Nm (PIP) per finger	Self-adjusting to finger size	-
Vanderbilt Hand Exoskeleton, (Gasser et al., 2017)	Assistive exoskeleton with simultaneous actuation of index to litte finger	1 / 1	-	50 (peak)	Designed for 50th percentile male hand	-
FINGER, (Taheri et al., 2014)	Grounded, highly backdrivable exoskeleton	2 / 2	-	-	Adjustable lengths of linkages	-
(Yap et al., 2016)	Soft robotic glove for assisted finger extension	- / 5	-	4.25 Nm (extension only)	-	-
HEXORR, (Schabowsky et al., 2010)	Grounded, backdrivable exoskeleton with force assistance mode	2 / 2	0-90 (MCP), 0-90 (PIP)[3]	-	Adjustable lengths of linkages	-

[1] Measured / acutated degrees of freedom. [2] Range of motion [3] The ranges of motion or forces for some degrees of freedom are missing in the table, see reference for additional information.

Device	Description	DoF[1]	RoM[2] (°)	Force (N)	Setup & adjustments	Haptic rendering
BiomHED, (Lee et al., 2014)	Biomimetic soft robotic glove for grasping assistance	- / 4	-	-	Zipper on palmar side facilitates donning	-
(Ueki et al., 2012)	Multi-degree-of-freedom device for assisted hand and wrist rehabilitation	18 / 18	-	0.5Nm (MCP finger), 1.8Nm (CMC thumb) [3]	Adjustable to hand size	-
(Xu et al., 2020)	Underactuated cable-driven soft robotic glove	3 / 1	-	35	-	-
HIRO-III, (Hioki et al., 2011)	Multi-fingered haptic end-effector interface	15 / 15	-	3.6 per finger	One size fits all, magnetic finger holders required	yes
(Yasuhisa Hasegawa et al., 2008)	Exoskeleton for grasping assistance	8 / 8	-	5	-	-
TenoExo, (Bützer et al., 2020)	Light-weight exoskeleton for grasping assistance	2 / 2	-15-90 (fingers overall), -10-45 (thumb overall)	6	Different sizes available	-
Gloreha, (Borboni et al., 2016)	Soft robotic glove with remote pneumatic actuation	5 / 5	-	-	Different sizes available	-
GripAble, (GripAble, UK)	Portable sensorized device for grasp training and grip strength assessment	1 / 0	-	-	One size fits all	-
HandSOME, (Brokaw et al., 2011)	Spring-actuated device for assisted finger extension training	0 / 0	0-90 (MCP fingers), 0-52 (CMC thumb)	4 Nm	Adjustable to hand size	-
HandCARE, (Dovat et al., 2008)	Rehabilitation system for cable-actuated finger training	5 / 5	0-70 (MCP)	15 per finger	Adjustable to hand size	yes

[1] Measured / acutated degrees of freedom. [2] Range of motion [3] The ranges of motion or forces for some degrees of freedom are missing in the table, see reference for additional information.

Device	Description	DoF[1]	RoM[2] (°)	Force (N)	Setup & adjustments	Haptic rendering
iHandRehab, (Li et al., 2011)	Interactive exoskeleton for active and passive rehabilitation	20 / 20	0-84 (MCP), 0-102 (PIP), 0-47 (DIP)[3]	-	Adjustable by fastening screws	yes
Maestro, (Yun et al., 2017)	Impedance-controlled cable-actuated exoskeleton	8 / 8	-	-	Adjustable lengths of linkages	-
(Just et al., 2019)	Hand module for ARMin upper-limb exoskeleton	1 / 1	-	200	One size fits all	-
Inmotion Hand, (BIONIK Laboratories, Canada)	Hand module for Inmotion ARM end-effector robot	1 / 1	-	-	Adjustable to hand size	-
(Frisoli et al., 2007)	Haptic interface for index finger and thumb with potential application in rehabilitation	6 / 6	-	4	-	yes
KULEX-Hand, (Hong et al., 2019)	Underactuated index finger and thumb exoskeleton for grasping assistance	1 / 1	-	10	-	-
Mano, (Randazzo et al., 2018)	Light-weight exoskeleton for grasping assistance	5 / 5	-	5 per finger	-	-
ManovoPower, (Hocoma, Switzerland)	Hand module for ArmeoPower upper-limb exoskeleton	1 / 1	-	-	One size fits all	-
Graspy Glove, (Popov et al., 2017)	Soft robotic glove for grasping assistance	4 / 4	-	16	Adjustable straps, soft structure	-
(Birouaş et al., 2020)	Underactuated exoskeleton	4 / 4	-	-	Customized parts required for each hand size	-

[1] Measured / acutated degrees of freedom. [2] Range of motion [3] The ranges of motion or forces for some degrees of freedom are missing in the table, see reference for additional information.

Device	Description	DoF[1]	RoM[2] (°)	Force (N)	Setup & adjustments	Haptic rendering
Gentle / G, (Loureiro and Harwin, 2007)	Hand module of the Gentle / G reach and grasp therapy system	3 / 3	0-70 (MCP), 0-90 (PIP), 10-60 (MCP thumb)	14 (fingers) / 12 (thumb)	Adjustable lengths of linkages, hinge mechanism for quick setup	yes
ReachMAN2, (Zhu et al., 2014)	Grounded device for arm reaching and grasping training	3 / 3	10-110 (fingers overall)	1.5 Nm	Height-adjustable for subjects in wheelchair	yes
SAFE, (Ben-Tzvi and Ma, 2015)	Underactuated exoskeleton with force sensing and haptic rendering	6 / 6	-30-90 (MCP), 0-100 (PIP), -5-70 (DIP)[3]	10 per finger	Designed for medium size, adjustable/ deformable fingertip fixation	yes
DexoHand, (Pu et al., 2016)	Grounded cable-driven exoskeleton	5 / 5	-5-75 (MCP), 0-90 (PIP), 0-60 (DIP)[3]	-	-	-
PLUTO, (Nehrujee et al., 2021)	Grounded robotic device with multiple exchangeable end-effectors for hand and forearm rehabilitation	1 / 1	12 cm[3]	55[3]	One size fits all, fingers are clamped to the handle	-

[1] Measured / acutated degrees of freedom. [2] Range of motion [3] The ranges of motion or forces for some degrees of freedom are missing in the table, see reference for additional information.

REFERENCES

Alnajjar, F., Umari, H., Ahmed, W. K., Gochoo, M., Vogan, A. A., Aljumaily, A., et al. (2021). CHAD: Compact Hand-Assistive Device for enhancement of function in hand impairments[Formula presented]. *Robotics and Autonomous Systems* 142, 103784. doi:10.1016/j.robot.2021.103784

Ates, S., Mora-Moreno, I., Wessels, M., and Stienen, A. H. (2015). Combined active wrist and hand orthosis for home use: Lessons learned. *IEEE International Conference on Rehabilitation Robotics* 2015-Septe, 398–403. doi:10.1109/ICORR.2015.7281232

Ben-Tzvi, P. and Ma, Z. (2015). Sensing and Force-Feedback Exoskeleton (SAFE) Robotic Glove. *IEEE Transactions on Neural Systems and Rehabilitation Engineering* 23, 992–1002. doi:10.1109/TNSRE.2014.2378171

BIONIK Laboratories (2021). *InMotion ARM/HAND,*. `https://www.bioniklabs.com/products/inmotion-arm-hand/` [Accessed February 27, 2021]

Birouaş, F. I., Ţarcă, R. C., Dzitac, S., and Dzitac, I. (2020). Preliminary results in testing of a novel asymmetric underactuated robotic hand exoskeleton for motor impairment rehabilitation. *Symmetry* 12. doi:10.3390/sym12091470

Borboni, A., Mor, M., and Faglia, R. (2016). Gloreha-Hand Robotic Rehabilitation: Design, Mechanical Model, and Experiments. *Journal of Dynamic Systems, Measurement and Control, Transactions of the ASME* 138. doi:10.1115/1.4033831

Brokaw, E. B., Black, I., Holley, R. J., and Lum, P. S. (2011). Hand Spring Operated Movement Enhancer (HandSOME): A portable, passive hand Exoskeleton for stroke rehabilitation. *IEEE Transactions on Neural Systems and Rehabilitation Engineering* 19, 391–399. doi:10.1109/TNSRE.2011.2157705

Bützer, T., Lambercy, O., Arata, J., and Gassert, R. (2020). Fully Wearable Actuated Soft Exoskeleton for Grasping Assistance in Everyday Activities. *Soft Robotics* 00, soro.2019.0135. doi:10.1089/soro.2019.0135

Cheng, L., Chen, M., and Li, Z. (2018). Design and Control of a Wearable Hand Rehabilitation Robot. *IEEE Access* 6, 74039–74050. doi:10.1109/ACCESS.2018.2884451

CyberGlove Systems (2021). *CyberGrasp,*. `http://www.cyberglovesystems.com/cybergrasp` [Accessed July 22, 2021]

Decker, M. and Kim, Y. (2017). A hand exoskeleton device for robot assisted sensory-motor training after stroke. *2017 IEEE World Haptics Conference, WHC 2017* , 436–441doi:10.1109/WHC.2017.7989941

Dovat, L., Lambercy, O., Gassert, R., Maeder, T., Milner, T., Teo Chee Leong, et al. (2008). HandCARE : A Cable-Actuated Rehabilitation System to Train Hand Function After Stroke. *IEEE Transactions on Neural Systems and Rehabilitation Engineering* 16, 582–591. doi:10.1109/TNSRE.2008.2010347

Frisoli, A., Simoncini, F., Bergamasco, M., and Salsedo, F. (2007). Kinematic Design of a Two Contact Points Haptic Interface for the Thumb and Index Fingers of the Hand 129. doi:10.1115/1.2712219

Gasser, B. W., Bennett, D. A., Durrough, C. M., and Goldfarb, M. (2017). Design and preliminary assessment of Vanderbilt hand exoskeleton. *IEEE International Conference on Rehabilitation Robotics* , 1537–1542doi:10.1109/ICORR.2017.8009466

GripAble (2021). *GripAble,*. `https://gripable.co/` [Accessed July 22, 2021]

Hesse, S., Kuhlmann, H., Wilk, J., Tomelleri, C., and Kirker, S. G. (2008). A new electromechanical trainer for sensorimotor rehabilitation of paralysed fingers: A case series in chronic and acute stroke patients. *Journal of NeuroEngineering and Rehabilitation* 5, 2–7. doi:10.1186/1743-0003-5-21

Hioki, M., Kawasaki, H., Sakaeda, H., Nishimoto, Y., and Mouri, T. (2011). Finger Rehabilitation Support System Using a Multifingered Haptic Interface Controlled by a Surface Electromyogram. *Journal of*

Robotics 2011, 1–10. doi:10.1155/2011/167516

Ho, N. S. K., Tong, K. Y., Hu, X. L., Fung, K. L., Wei, X. J., Rong, W., et al. (2011). An EMG-driven exoskeleton hand robotic training device on chronic stroke subjects: Task training system for stroke rehabilitation. In *2011 IEEE International Conference on Rehabilitation Robotics* (IEEE), March 2011, 1–5. doi:10.1109/ICORR.2011.5975340

Hocoma (2021). *ManovoPower,*. `https://www.hocoma.com/solutions/armeo-power/modules/` [Accessed April 04, 2021]

Hong, M. B., Kim, S. J., Ihn, Y. S., Jeong, G.-C., and Kim, K. (2019). KULEX-Hand: An Underactuated Wearable Hand for Grasping Power Assistance. *IEEE Transactions on Robotics* 35, 420–432. doi:10.1109/TRO.2018.2880121

Huang, H., Zhu, A., Song, J., Tu, Y., Shi, X., and Guo, Z. (2020). Characterization and Evaluation of A Cable-Actuated Flexible Hand Exoskeleton. *2020 17th International Conference on Ubiquitous Robots, UR 2020* , 56–61doi:10.1109/UR49135.2020.9144871

Just, F., Gunz, D., Duarte, J., Simonetti, D., Riener, R., and Rauter, G. (2019). Improving Usability of Rehabilitation Robots: Hand Module Evaluation of the ARMin Exoskeleton (Cham: Springer International Publishing), vol. 22 of *Biosystems & Biorobotics*. 80–84. doi:10.1007/978-3-030-01887-0_16

Kim, D. H. and Park, H.-S. (2018). Cable Actuated Dexterous (CADEX) Glove for Effective Rehabilitation of the Hand for Patients with Neurological diseases. In *2018 IEEE/RSJ International Conference on Intelligent Robots and Systems (IROS)* (IEEE), 2305–2310. doi:10.1109/IROS.2018.8594336

Lee, S. W., Landers, K. A., and Park, H. S. (2014). Development of a biomimetic hand exotendon device (BiomHED) for restoration of functional hand movement post-stroke. *IEEE Transactions on Neural Systems and Rehabilitation Engineering* 22, 886–898. doi:10.1109/TNSRE.2014.2298362

Leonardis, D., Barsotti, M., Loconsole, C., Solazzi, M., Troncossi, M., Mazzotti, C., et al. (2015). An EMG-controlled robotic hand exoskeleton for bilateral rehabilitation. *IEEE Transactions on Haptics* 8, 140–151. doi:10.1109/TOH.2015.2417570

Li, J., Zheng, R., Zhang, Y., and Yao, J. (2011). iHandRehab: An interactive hand exoskeleton for active and passive rehabilitation. *IEEE International Conference on Rehabilitation Robotics* , 1–6doi:10.1109/ICORR.2011.5975387

Loureiro, R. C. and Harwin, W. S. (2007). Reach & Grasp Therapy: Design and Control of a 9-DOF Robotic Neuro-rehabilitation System. In *2007 IEEE 10th International Conference on Rehabilitation Robotics* (IEEE), vol. 00, 757–763. doi:10.1109/ICORR.2007.4428510

Marconi, D., Baldoni, A., McKinney, Z., Cempini, M., Crea, S., and Vitiello, N. (2019). A novel hand exoskeleton with series elastic actuation for modulated torque transfer. *Mechatronics* 61, 69–82. doi:10.1016/j.mechatronics.2019.06.001

Masia, L., Krebs, H. I., Cappa, P., and Hogan, N. (2007). Design and Characterization of Hand Module for Whole-Arm Rehabilitation Following Stroke. *IEEE/ASME Transactions on Mechatronics* 12, 399–407. doi:10.1109/TMECH.2007.901928

Metzger, J.-C., Lambercy, O., Chapuis, D., and Gassert, R. (2011). Design and characterization of the ReHapticKnob, a robot for assessment and therapy of hand function. *2011 IEEE/RSJ International Conference on Intelligent Robots and Systems* , 3074–3080doi:10.1109/iros.2011.6094882

Nehrujee, A., Andrew, H., Reethajanetsurekha, Patricia, A., Samuelkamaleshkumar, S., Prakash, H., et al. (2021). Plug-and-Train Robot (PLUTO) for Hand Rehabilitation: Design and Preliminary Evaluation. *IEEE Access* 9, 134957–134971. doi:10.1109/ACCESS.2021.3115580

Popov, D., Gaponov, I., and Ryu, J. H. (2017). Portable exoskeleton glove with soft structure for hand assistance in activities of daily living. *IEEE/ASME Transactions on Mechatronics* 22, 865–875. doi:10.1109/TMECH.2016.2641932

Proxima Medical Technology (2021). *ArmAssist,*. `http://www.armassist.eu/` [Accessed January 02, 2020]

Pu, S. W., Chang, J. Y., Pei, Y. C., Kuo, C. C., and Wang, M. J. (2016). Anthropometry-based structural design of a hand exoskeleton for rehabilitation. *M2VIP 2016 - Proceedings of 23rd International Conference on Mechatronics and Machine Vision in Practice* doi:10.1109/M2VIP.2016.7827282

Pu, S. W., Pei, Y. C., and Chang, J. Y. (2020). Decoupling finger joint motion in an exoskeletal hand: A design for robot-assisted rehabilitation. *IEEE Transactions on Industrial Electronics* 67, 686–697. doi:10.1109/TIE.2019.2912793

Randazzo, L., Iturrate, I., Perdikis, S., and Millán, J. D. (2018). Mano: A Wearable Hand Exoskeleton for Activities of Daily Living and Neurorehabilitation. *IEEE Robotics and Automation Letters* 3, 500–507. doi:10.1109/LRA.2017.2771329

Rudd, G., Daly, L., Jovanovic, V., and Cuckov, F. (2019). A low-cost soft robotic hand exoskeleton for use in therapy of limited hand-motor function. *Applied Sciences (Switzerland)* 9. doi:10.3390/app9183751

Sandoval-Gonzalez, O., Jacinto-Villegas, J., Herrera-Aguilar, I., Portillo-Rodiguez, O., Tripicchio, P., Hernandez-Ramos, M., et al. (2016). Design and development of a hand exoskeleton robot for active and passive rehabilitation. *International Journal of Advanced Robotic Systems* 13. doi:10.5772/62404

Sarac, M., Solazzi, M., Sotgiu, E., Bergamasco, M., and Frisoli, A. (2016). Design and kinematic optimization of a novel underactuated robotic hand exoskeleton. *Meccanica* 52, 749–761. doi:10.1007/s11012-016-0530-z

Schabowsky, C. N., Godfrey, S. B., Holley, R. J., and Lum, P. S. (2010). Development and pilot testing of HEXORR: Hand EXOskeleton Rehabilitation Robot. *Journal of NeuroEngineering and Rehabilitation* 7, 36. doi:10.1186/1743-0003-7-36

Sooraj, R., Akshay, N., Jeevan, T. G., and Bhavani, R. R. (2013). Design and analysis of a parallel haptic orthosis for upper limb rehabilitation. *International Journal of Engineering and Technology* 5, 444–451

Taheri, H., Rowe, J. B., Gardner, D., Chan, V., Gray, K., Bower, C., et al. (2014). Design and preliminary evaluation of the FINGER rehabilitation robot: controlling challenge and quantifying finger individuation during musical computer game play. *Journal of NeuroEngineering and Rehabilitation* 11, 10. doi:10.1186/1743-0003-11-10

Takahashi, C. D., Der-Yeghiaian, L., Le, V. H., and Cramer, S. C. (2005). A robotic device for hand motor therapy after stroke. *Proceedings of the 2005 IEEE 9th International Conference on Rehabilitation Robotics* 2005, 17–20. doi:10.1109/ICORR.2005.1501041

TyroMotion (2021). *Amadeo,*. `https://tyromotion.com/en/products/amadeo/` [Accessed July 22, 2021]

Ueki, S., Kawasaki, H., Ito, S., Nishimoto, Y., Abe, M., Aoki, T., et al. (2012). Development of a Hand-Assist Robot With Multi-Degrees-of-Freedom for Rehabilitation Therapy. *IEEE/ASME Transactions on Mechatronics* 17, 136–146. doi:10.1109/TMECH.2010.2090353

Wang, D., Meng, Q., Meng, Q., Li, X., and Yu, H. (2018). Design and Development of a Portable Exoskeleton for Hand Rehabilitation. *IEEE Transactions on Neural Systems and Rehabilitation Engineering* 26, 2376–2386. doi:10.1109/TNSRE.2018.2878778

Xu, D., Wu, Q., and Zhu, Y. (2020). Development of a soft cable-driven hand exoskeleton for assisted rehabilitation training. *Industrial Robot* 48, 189–198. doi:10.1108/IR-06-2020-0127

Yang, J., Xie, H., and Shi, J. (2016). A novel motion-coupling design for a jointless tendon-driven finger exoskeleton for rehabilitation. *Mechanism and Machine Theory* 99, 83–102. doi:10.1016/j.mechmachtheory.2015.12.010

Yap, H. K., Lim, J. H., Goh, J. C. H., and Yeow, C.-H. (2016). Design of a Soft Robotic Glove for Hand Rehabilitation of Stroke Patients With Clenched Fist Deformity Using Inflatable Plastic Actuators. *Journal of Medical Devices* 10, 5–10. doi:10.1115/1.4033035

Yasuhisa Hasegawa, Yasuyuki Mikami, Kosuke Watanabe, and Yoshiyuki Sankai (2008). Five-fingered assistive hand with mechanical compliance of human finger. In *2008 IEEE International Conference on Robotics and Automation* (IEEE), 718–724. doi:10.1109/ROBOT.2008.4543290

Yun, Y., Dancausse, S., Esmatloo, P., Serrato, A., Merring, C. A., Agarwal, P., et al. (2017). Maestro: An EMG-driven assistive hand exoskeleton for spinal cord injury patients. In *2017 IEEE International Conference on Robotics and Automation (ICRA)* (IEEE), 2904–2910. doi:10.1109/ICRA.2017.7989337

Zbytniewska, M., Rinderknecht, M. D., Lambercy, O., Barnobi, M., Raats, J., Lamers, I., et al. (2019). Design and Characterization of a Robotic Device for the Assessment of Hand Proprioceptive, Motor, and Sensorimotor Impairments. In *2019 IEEE 16th International Conference on Rehabilitation Robotics (ICORR)* (IEEE), 441–446. doi:10.1109/ICORR.2019.8779507

Zhang, F., Hua, L., Fu, Y., Chen, H., and Wang, S. (2014). Design and development of a hand exoskeleton for rehabilitation of hand injuries. *Mechanism and Machine Theory* 73, 103–116. doi:10.1016/j.mechmachtheory.2013.10.015

Zhu, T. L., Klein, J., Dual, S. A., Leong, T. C., and Burdet, E. (2014). ReachMAN2: A compact rehabilitation robot to train reaching and manipulation. *IEEE International Conference on Intelligent Robots and Systems* , 2107–2113doi:10.1109/IROS.2014.6942845
